# Single-cell analysis reveals the origins and intrahepatic development of liver-resident IFN-γ-producing γδ T cells

**DOI:** 10.1038/s41423-021-00656-1

**Published:** 2021-03-10

**Authors:** Yuan Hu, Keke Fang, Yanan Wang, Nan Lu, Haoyu Sun, Cai Zhang

**Affiliations:** 1 Institute of Immunopharmaceutical Sciences, School of Pharmaceutical Sciences, Cheeloo College of Medicine, Shandong University, Jinan, Shandong China; 2 Institute of Diagnostics, School of Medicine, Cheeloo College of Medicine, Shandong University, Jinan, Shandong China; 3 Institute of Immunology, School of Basic Medical Sciences, Division of Life Sciences and Medicine, University of Science and Technology of China, Hefei, Anhui China

**Keywords:** γδ T cells, liver, extrathymic development, subpopulation, differentiation, Lymphocytes, Cell biology

## Abstract

γδ T cells are heterogeneous lymphocytes located in various tissues. However, a systematic and comprehensive understanding of the origins of γδ T cell heterogeneity and the extrathymic developmental pathway associated with liver γδ T cells remain largely unsolved. In this study, we performed single-cell RNA sequencing (scRNA-seq) to comprehensively catalog the heterogeneity of γδ T cells derived from murine liver and thymus samples. We revealed the developmental trajectory of γδ T cells and found that the liver contains γδ T cell precursors (pre-γδ T cells). The developmental potential of hepatic γδ T precursor cells was confirmed through in vitro coculture experiments and in vivo adoptive transfer experiments. The adoptive transfer of hematopoietic progenitor Lin^−^Sca-1^+^Mac-1^+^ (LSM) cells from fetal or adult liver samples to sublethally irradiated recipients resulted in the differentiation of liver LSM cells into pre-γδ T cells and interferon-gamma^+^ (IFN-γ^+^) but not interleukin-17a^+^ (IL-17a^+^) γδ T cells in the liver. Importantly, thymectomized mouse models showed that IFN-γ-producing γδ T cells could originate from liver LSM cells in a thymus-independent manner. These results suggested that liver hematopoietic progenitor LSM cells were able to differentiate into pre-γδ T cells and functionally mature γδ T cells, which implied that these cells are involved in a distinct developmental pathway independent of thymus-derived γδ T cells.

## Introduction

γδ T cells are a unique group of lymphocytes that display immunologic features that are common to both the innate and adaptive immune systems. γδ T cells predominantly reside in mucosal and epithelial barriers and other peripheral tissues and respond rapidly during infection by exerting potent cytotoxic effects and producing cytokines, functioning as the first line of immune defense against infections.^1–3^ γδ T cells also regulate the activation, migration, and effector functions of other immune cells by producing cytokines and chemokines. γδ T cells play crucial roles in antitumor immune responses, exerting direct cytotoxic effects to eliminate tumor cells or indirectly modulating the activities and functions of other immune cells.^4,5^ The functional plasticity of γδ T cells depends on the developmental program in the thymus and functional polarization in the periphery and correlates with γδ T cell subpopulations found in various tissue locations.^6^ Several γδ T cell subsets establish preferential residency in particular tissues.^7,8^ However, to date, a systematic and comprehensive understanding of γδ T cell heterogeneity remains lacking.

The thymus is the primary site for the development, differentiation, and maturation of γδ T cells. Unlike conventional αβ T cells, which develop in the thymus in a naïve state and acquire effector functions in the periphery, most γδ T cells acquire their effector fates during development in the thymus. However, a growing body of evidence has indicated that some γδ T cells conserve plasticity and can differentiate into functional subpopulations in the periphery.^6,9^ Extrathymic developmental programs appear to exist for γδ T cells, particularly intestinal intraepithelial γδ (γδ IEL) T cells, which can develop locally in gut cryptopatches.^10^ The adult liver contains hematopoietic stem and progenitor cells (HSPCs) and is considered to serve as an extramedullary hematopoietic organ. C-kit^+^ stem cells derived from the adult mouse liver have been reported to generate γδ IEL T cells in irradiated severe combined immunodeficient mice.^11^ Adult liver HSPCs have also been reported to differentiate into T and B lymphocytes.^12^ More recent studies have focused on the developmental origins of γδ T cells and related transcriptional programs.^13,14^ However, the precise developmental program, particularly the origins and extrathymic developmental pathways associated with liver γδ T cells, remains largely unsolved.

In this study, we performed single-cell RNA sequencing (scRNA-seq) to comprehensively catalog the heterogeneity of γδ T cells derived from murine liver and thymus samples. We identified 4 clusters and 6 clusters of γδ T cells in the thymus and liver, respectively. Importantly, we observed that the liver contains γδ T precursor cells (pre-γδ T cells) and further defined the developmental trajectory of these γδ T cell subsets using a pseudotemporal reconstruction approach. We next evaluated the developmental potential of hepatic γδ T precursor cells using an in vitro coculture experiment and an in vivo adoptive transfer experiment. Moreover, we explored the possibility that hepatic γδ T cells might develop from liver HSPCs. We found that liver hematopoietic progenitor Lin^−^Sca-1^+^Mac-1^+^ (LSM) cells were able to differentiate into pre-γδ T cells and functionally mature γδ T cells in a thymus-independent manner, which implied a distinct developmental pathway independent of thymus-derived γδ T cells.

## Materials and methods

### Animal strains

Male (6–8 weeks old) and pregnant C57BL/6 J (B6, *CD45.2*^*+*^) mice were obtained from Hua Fukang Biological Technology Co., Ltd. (Beijing, China). C57BL/6-Ly5.1 (*CD45.1*^*+*^) mice were obtained from Beijing Vital River Laboratory Animal Technology Co., Ltd. (Beijing, China). C57BL/6J-derived T-cell receptor δ (TCRδ) knockout (*TCRδ*^*−/−*^) mice (*TCRδ*^*−/−*^, *CD45.2*^*+*^) were obtained from The Jackson Laboratory (Bar Harbor, ME, USA). *CD45.1*^*+*^ and *TCRδ*^*−/−*^ mice were bred in our laboratory under specific pathogen-free conditions. All mice were maintained under specific pathogen-free conditions. Experiments were performed according to the guidelines for experimental animals from Shandong University and were approved by the Committee on the Ethics of Animal Experiments of Shandong University.

### OP9-DL1 coculture

For short-term cultures of sorted pre-γδ T cells, OP9-DL1 coculture assays were performed as previously described.^15^ In brief, a total of 1 × 10^4^ CD24^+^CD73^+^CD3e^+^γδ TCR^+^ pre-γδ T cells were sorted from either liver or thymus samples, seeded on a monolayer of OP9-DL1 feeder cells, and maintained in α-minimal essential medium supplemented with 10% heat-inactivated fetal bovine serum, interleukin-7 (IL-7, 1 ng/mL) and FMS-like tyrosine kinase 3 ligand (FLT3-L, 5 ng/mL). Sorted pre-γδ T cells were transferred into 96-well plates containing new OP9-DL1 feeder cells every 3 days. The cocultured cells were harvested on days 3, 5, and 9. Antibody staining and flow cytometry were performed on the harvested cells.

### In vitro polarization culture

The plasticity of pre-γδ T cells was assessed by performing in vitro polarization assays, as previously described.^16^ CD24^+^CD73^+^ pre-γδ T cells from thymus and liver samples and CD44^lo^ naïve γδ T cells from lymph nodes were sorted by flow cytometry, followed by stimulation for 4 days with coated anti-CD3 (clone 145.2C11) and anti-CD28 (clone 37.51) antibodies, both at 4 μg/mL; for T-helper cell (Th1)-like conditions, human IL-2 (13 ng/mL) and mouse IL-12 (10 ng/mL) were included; for Th17-like conditions, anti-interferon (IFN)-γ (clone R4-6A2) neutralizing antibody (5 μg/mL), mouse IL-6 (20 ng/mL), mouse IL-23 (10 ng/mL), and human transforming growth factor (TGF)-β1 (10 ng/mL) were included; and for inducible T-regulatory (iTreg)-like conditions, human IL-2 (13 ng/mL) and human TGF-β1 (10 ng/mL) were included.

### Flow cytometry

Mononuclear cells (MNCs) were harvested from the liver, spleen, thymus, and small intestine in mice. Single-cell suspensions were blocked with an anti-mouse CD16/CD32 antibody (clone, 2.4G2) at room temperature for 10 min. The cells were then stained with a cocktail of antibodies (mAb details are described in Supplemental Table 1) to identify surface molecules. After 30 min of staining at room temperature, the cells were washed twice with a 1× phosphate buffer solution. Flow cytometric analyses were performed on a fluorescence-activated cell sorter Aria III instrument (BD Biosciences) or a Beckman Coulter Gallios flow cytometer (Beckman Coulter). Data were analyzed using FlowJo software (Treestar, Inc., Ashland, OR, USA).

Cytokine production was assessed ex vivo by intracellular staining after stimulation with phorbol 12-myristate 13-acetate (PMA, 30 ng/mL, Sigma–Aldrich) and ionomycin (1 µg/mL, Sigma–Aldrich) for 4 h. Brefeldin A and monensin were added to the culture after 1 h of stimulation. After surface staining, the cells were fixed, permeabilized, and stained with the mAbs against the indicated intracellular molecules (Supplemental Table 1).

### Cell sorting

Hepatic or thymic MNCs were stained with anti-CD3e, anti-TCRγ/δ, anti-CD24, and anti-CD73 antibodies to sort CD24^+^CD73^+^CD3e^+^γδ TCR^+^ pre-γδ T cells. Hepatic MNCs were stained with a lineage antibody cocktail (including antibodies against CD3, CD19, NK1.1, Gr1, Ter119, and CD11c) to sort Lin^−^ cells. Hepatic MNCs were incubated with the lineage antibody cocktail, anti-CD11b (Mac-1), and anti-Sca-1 antibodies to sort Lin^−^Sca-1^+^Mac-1^+^ (LSM) cells. Sorting was performed with the fluorescence-activated cell sorter Aria III or the cell sorter MoFlo Astrios EQ (Beckman Coulter).

### Thymectomy and adoptive transfer

Thymectomy was performed on 5–6-week-old B6 or *TCRδ*^*−/−*^ mice and performed by Cyagen Biosciences, China. All operated mice were allowed to recover at least 3 weeks before adoptive transfer. *CD45.1*^*+*^, *TCRδ*^*−/−*^ recipient mice (6–8 weeks old) or thymectomized mice were sublethally irradiated with 5 Gy one day prior to adoptive transfer and maintained on antibiotic-containing water (neomycin sulfate at 2 g/L) for at least 2 weeks. The day after irradiation, 1 × 10^6^ MNCs isolated from the thymus or liver at embryonic day 16.5 were adoptively transferred into *CD45.1*^*+*^ mice by tail intravenous injection. A total of 3 × 10^4^ pre-γδ T cells, 1 × 10^5^ Lin^−^ cells, or 1–5 × 10^4^ LSM cells were intrasplenically or intravenously transferred into the irradiated recipients.

### Parabiosis model

The parabiotic surgery was performed as previously described.^17^ In brief, a B6 (*CD45.2*^*+*^) mouse and a *CD45.1*^*+*^ mouse were selected of the same sex and similar weights. Mice were anesthetized, and the opposite flanks of each mouse were thoroughly shaved. The mice were placed back-to-back with the shaved areas adjacent. Longitudinal skin incisions were performed on the shaved sides of each mouse, starting 0.5 cm above the elbow and extending to 0.5 cm below the knee joint. The corresponding olecranon and knee joints of the mice were fixed together with nonabsorbable silk. Finally, the corresponding dorsal and ventral skin edges of each mouse were sutured together. Complete blood chimerism was established within 2–3 weeks.

### Cell preparation and single-cell RNA sequencing

Hepatic and thymic MNCs were harvested from B6 mice (8 weeks old). Thymic cell suspensions were enriched for γδ T cells by magnetic bead negative selection using anti-CD4 and anti-CD8 magnetic beads (Miltenyi Biotec). Cells were stained with anti-CD3e and anti-TCRγ/δ antibodies, as described above. After surface staining, hepatic or thymic γδ T cells were sorted using a MoFlo Astrios EQ. After sorting, the cells were washed and maintained on ice. ScRNA-Seq libraries were prepared using the 10× Genomics Chromium single-cell 3’ v2 kit, according to standard protocol provided by CapitalBio Technology Co., Ltd. (Beijing, China). RNA-seq was performed on an Illumina HiSeq X Ten system using 150 bp paired-end sequencing (PE150). All sequencing data have been submitted to the National Center for Biotechnology Information Gene Expression Omnibus (NCBI GEO) depository under the accession number GEO: GSE164106.

### Single-cell RNA-seq analysis

ScRNA-seq analyses were processed as previously described^18,19^ and were performed by CapitalBio Technology. In brief, the Seurat R package (v3.3.1) offers functions for quality control, filtering, and clustering. We filtered out any cells expressing fewer than 800 genes or more than 4000 genes per cell. Any cell with mitochondrial unique molecular identifier (UMI) counts greater than 6% or ribosomal UMI counts greater than 50% was also filtered out. A total of 5835 single cells from liver samples and 1790 single cells from thymus samples were analyzed together. The top 30 principal components were selected for t-distributed stochastic neighbor embedding (t-SNE) clustering analysis. A parameter resolution of 0.6 was selected to cluster 8 groups. The pseudotemporal ordering of 8 clusters was performed using Monocle 2.

### Gene ontology (GO) enrichment analysis

GO enrichment of cluster markers was performed using KOBAS software with the Benjamini-Hochberg multiple testing adjustment using the top 20 marker genes of the cluster. Enrichment scores (*P*-values) for selected numbers of GO annotations were calculated with a hypergeometrical statistical test with a threshold of 0.05. The data were plotted as -log10 of the *P*-value after Benjamini-Hochberg correction.

### Statistical analysis

The data were analyzed using GraphPad Prism 8 software (GraphPad Software, USA). Data are presented as the mean ± standard error of the mean (SEM). Differences among more than two groups were assessed by one-way analysis of variance (ANOVA), and differences between two groups were assessed by a two-tailed Student’s *t*-test. A *P*-value < 0.05 was considered significant (*P < 0.05; **P < 0.01; ***P < 0.001).

## Results

### Origins and developmental pathways associated with γδ T cells based on single-cell analysis

**Fig. 1:**
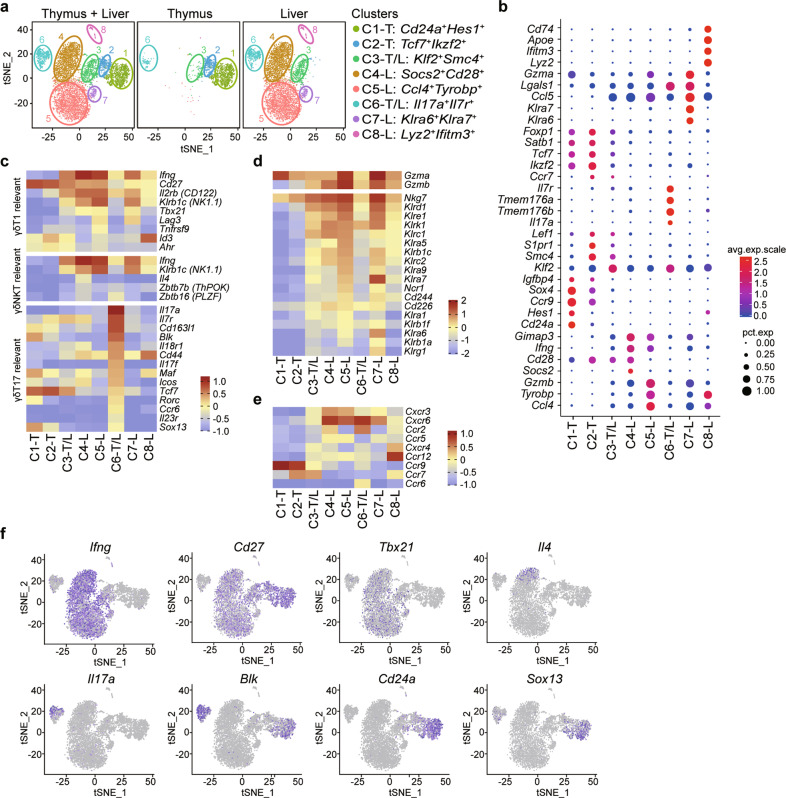
Single-cell RNA sequencing identified murine thymic and hepatic γδ T cell subpopulations. **a** Two-dimensional visualization of single-cell clusters in thymic and hepatic γδ T lymphocytes from 10 B6 mice (8 weeks old) by t-distributed stochastic neighbor embedding (t-SNE), showing the formation of 8 clusters, which are indicated by different colors. **b** Dot plot of 8 clusters with unique signature gene expression profiles. **c**–**e** Heatmap analysis of the relative expression levels of genes associated with γδ T1, γδ NKT or γδ T17 cells (**c**), natural killer (NK) cell receptors (**d**) and chemokine receptors (**e**). **f** T-SNE map of the specified genes

We first sorted thymic and hepatic γδ T cells using flow cytometry (the purity of the sorted cells is shown in Supplementary Fig. S1) and investigated the profiles and heterogeneity associated with γδ T cells derived from murine thymus and liver samples using 10× Genomics scRNA-seq.^19^ A total of eight distinct clusters, including 4 clusters in the thymus and 6 clusters in the liver, were identified by t-SNE analysis (Fig. 1a, Supplemental Table 2). According to the tissue distributions of the 8 clusters, we divided them into three classifications: cluster 1 (C1) and C2 were uniquely localized to the thymus and named C1-T and C2-T; C4, C5, C7, and C8 were uniquely localized to the liver and named C4-L, C5-L, C7-L, and C8-L; C3 and C6 were identified in both the thymus and liver and named C3-T/L and C6-T/L (Fig. 1a). By comparing differences in gene expression among the 8 clusters (Fig. 1b–f), we defined their cellular identities, associated with the elevated expression of known cell population-specific transcripts. C4-L, C5-L, and C7-L were identified by the high expression levels of *Ifng*, *Cd27, Il2rb* (CD122), and *Klrb1c* (NK1.1), suggesting that these clusters might be IFN-γ-producing γδ T cells (Fig. 1c). C5-L was characterized by higher levels of *Ccl4*, *Tyrobp*, and *Gzmb*, whereas C4-L was characterized by the specific expression of *Socs2*, *Cd28*, and *Gimap3* (Fig. 1b). C7-L was identified by the expression of *Klra6*, *Klra7, Ccl5, Lgals1*, and *Gzma* (Fig. 1b). In addition, C5-L and C7-L were enriched in genes encoding natural killer (NK) cell receptors, such as *Nkg7, Klrd1, Klre1, Klrk1, Klrc1*, and *Klrb1c*, as well as *Gzma* and *Gzmb*, similar to C4-L but to a lesser extent (Fig. 1d). The chemokine receptor genes associated with different clusters are shown in Fig. 1e. Notably, C4-L expressed not only high levels of *Ifng* and *Klrb1c* but also high levels of *Il4* and detectable levels of *Zbtb7b* (ThPOK) and *Zbtb16* (PLZF), which are associated with γδ NKT cells (Fig. 1c, f). Thus, we concluded that C5-L and C7-L represented cytotoxic γδ T cell subsets, whereas C4-L was likely a γδ NKT cell-like subset. Several genes associated with γδ T17 lymphocytes, such as *Il17a*, *Il17f, Il7r, Blk*, *Maf*, *Rorc*, and *Ccr6*, were enriched in C6-T/L (Fig. 1b–f), whereas *Lyz2*, *Ifitm3, Apoe*, *Cd74*, and *Ccr12* were enriched in C8-L (Fig. 1b, e). The highest expression levels of *Cd24a*, *Ccr9*, *Sox4, Hes1*, *Sox13*, and *Maf*, which are genes commonly associated with γδ T progenitor cells, were observed in C1-T cells (Fig. 1b, c, f), suggesting that C1-T cells represented γδ T progenitor cells. In addition to C1-T, C2-T and C3-T/L also expressed higher levels of *Cd24a*, *Sox4*, and *Tcf7* than the other clusters (Fig. 1b), suggesting that C2-T and C3-T/L might represent an intermediate stage in the development process, transforming from C1-T to another mature γδ T cell subpopulation.

**Fig. 2:**
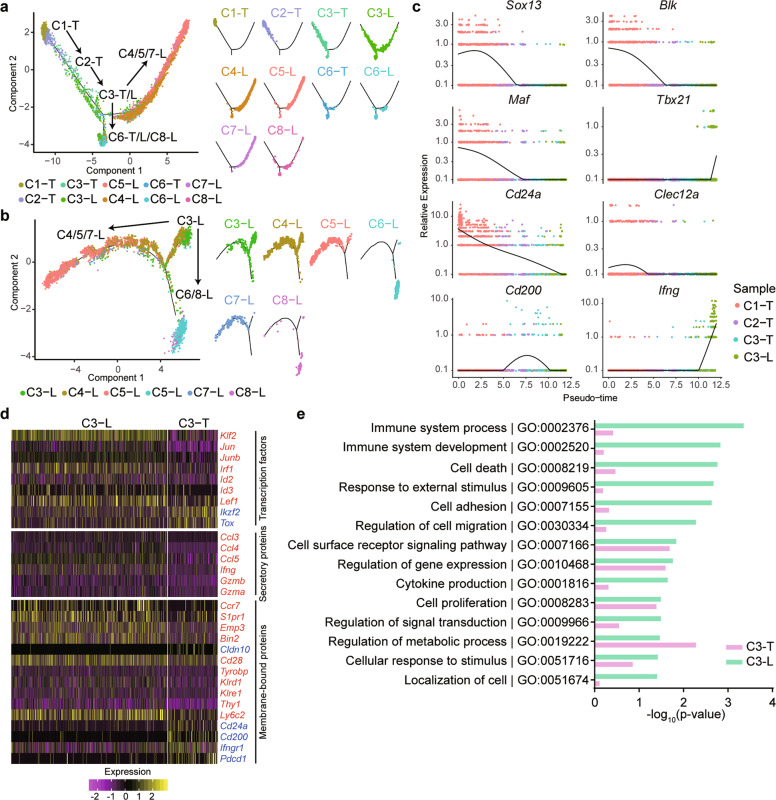
The developmental trajectory of murine γδ T cells identified by pseudotemporal reconstruction. **a** Pseudotemporal reconstruction of thymic and hepatic γδ T cell clusters. **b** Pseudotemporal reconstruction of hepatic γδ T cell clusters. **c** The expression trajectories of selected genes in pseudotemporal ordering among C1-T, C2-T, C3-T, and C3-L. **d** Heatmap showing the relative expression of select genes, including transcription factors and secretory and membrane-bound proteins, between the C3-T and C3-L clusters. Upregulated genes in the C3-L (red) or C3-T (blue) clusters are indicated. **e** Selected enriched GO terms comparing C3-L with C3-T

We found that the liver contained not only mature γδ T cell subpopulations, such as C4-L, C5-L, and C6-T/L but also contained the relatively rich transcriptional profile of C3-T/L (Fig. 1a), suggesting the existence of a liver-localized immature, developing γδ T precursor cell subset that may develop into mature γδ T cells locally. To further explore the developmental pathways of γδ T cells, a pseudotemporal maturation trajectory was reconstructed all thymic and hepatic γδ T cell clusters to visualize their developmental and differentiation trajectories (Fig. 2a). Three extremes were identified on the trajectory: C4/C5/C7-L on one end, C1-T on one end, and C6-T/L/C8-L on one end. These results suggested that C1-T represents an undifferentiated stage, whereas C4-L, C5-L, and C6-T/L locate at terminally differentiated stages based on their gene expression features (Fig. 1b–f); therefore, we speculated that C1-T represented the original γδ T progenitor cells. The next stage of the predicted trajectory is C2-T, followed by C3-T/L. Importantly, C3-T/L is located at the divergence point, indicating that the C3-T/L population represents the key differentiation point prior to the terminally differentiated stages. Both the thymus and liver contained C3-T/L clusters, but C1-T and C2-T were solely distributed to the thymus. To further determine whether the C3 population in the liver (C3-L) might represent a population of pre-γδ T cells for the intrahepatic differentiation of γδ T cells, a pseudotemporal maturation trajectory was reconstructed using transcripts that were only identified in liver γδ T cells (Fig. 2b). The pseudotemporal trajectory of hepatic γδ T cells showed C3-L located as the root of a tree, representing potentially pre-γδ T cells, whereas C4/5/7-L and C6/8-L established two separate branches of terminally differentiated γδ T cells (Fig. 2b). Therefore, we predicted that the developmental trajectory is C1-T → C2-T → C3-T/L → C4/5/7-L or C6-T/L/C8-L. To further verify the accuracy of the pseudotemporal differentiation order, the expression trajectories of genes that are known to be regulated during the developmental process of γδ T cells were analyzed (Fig. 2c and Supplementary Fig. S2). The results showed that the expression levels of *Sox13*, *Blk*, *Maf*, and *Cd24a* gradually reduced along the pseudotemporal progression (Fig. 2c), which was in line with the reported results. The expression levels of *Tbx21* and *Ifng* were upregulated in C3-L cells (Fig. 2c).

We further compared differences in gene expression between the C3 clusters in the thymus (C3-T) and liver (C3-L). As shown in Fig. 2d, the expression levels of 28 genes, including *Ikzf2*, *Tox*, *Cldn10*, *Cd24a Cd200*, *Ifngr1*, and *Pdcd1*, were higher in C3-T than in C3-L, whereas the levels of 55 genes, such as *Klf2*, *Irf1*, *Ccl5*, *Ifng*, *Ccr7*, *S1pr1*, and *Ly6c2*, were higher in C3-L than in C3-T. GO enrichment analysis indicated that C3-T was enriched in genes involved in the regulation of metabolic processes (Fig. 2e). In contrast, C3-L was specifically enriched in genes associated with immune system processes, immune system development, cytokine production, and cellular localization (Fig. 2e). We speculated that C3 precursor cells (pre-γδ T cells) in thymus might migrate to the liver, where they further develop into terminally differentiated γδ T cells, such as the γδ T1, γδ NKT, and γδ T17 subpopulations. The observed differences between the two C3 clusters might be due to different origins or to differences in the tissue microenvironments, which might modulate the gene expression profiles.

### Revealing liver-resident γδ T precursors cells

**Fig. 3:**
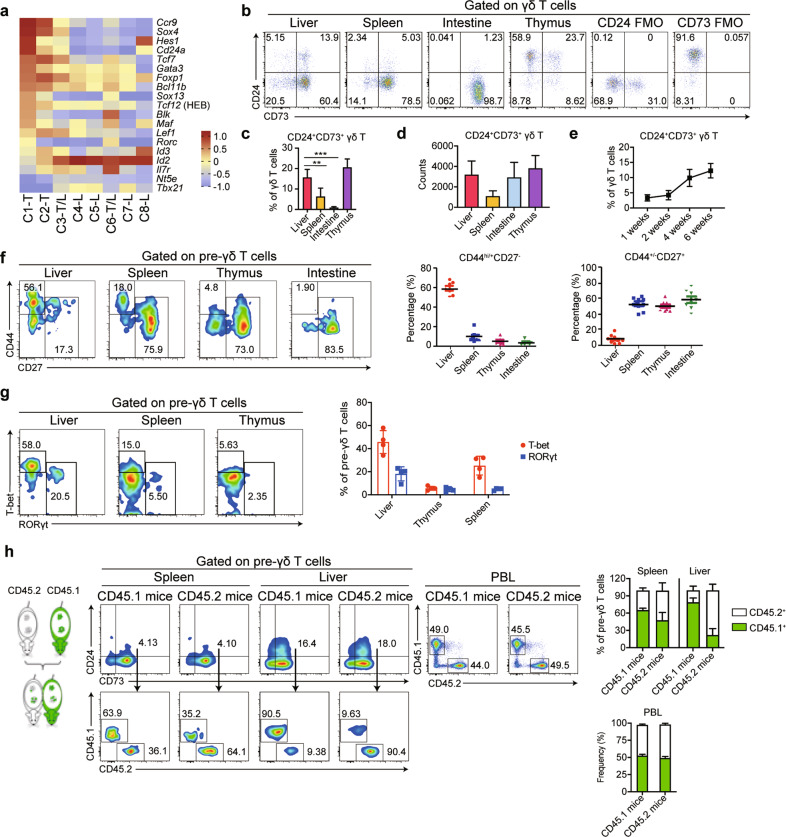
Liver contains tissue-resident γδ T precursors. **a** Heatmap analysis showing the relative expression of genes associated with γδ T progenitor cells. **b**, **c** The percentages of CD24^+^CD73^+^ pre-γδ T cells among total γδ T cells from the liver, spleen, intestine, and thymus detected by flow cytometry (6–8-week-old mice, *n* = 4). **d** The absolute numbers of pre-γδ T cells in tissues (6–8-week-old mice, *n* = 4–6). **e** Changes in hepatic pre-γδ T cell proportions among total γδ T cells with age (*n* = 4–6). **f** Flow cytometric plots showing the expression of CD27 and CD44 on pre-γδ T lymphocytes derived from the liver, spleen, thymus, and intestine (6-week-old mice, *n* = 7). **g** The expression of the transcription factors T-bet and RORγt in pre-γδ T cells was examined by flow cytometry (6-week-old mice, *n* = 3–4). **h** A B6 mouse (*CD45.2*^*+*^, 6–8 weeks old) and a *CD45.1*^*+*^ mouse (6–8 weeks old) were sutured together to establish the parabiosis model for two weeks. The percentages of CD45.1^+^ and CD45.2^+^ cells in peripheral blood lymphocytes (PBLs) were detected to verify whether the parabiosis models were successfully established. Flow cytometric analysis further identified the host origins (CD45.1^+^ or CD45.2^+^) of splenic or hepatic pre-γδ T cells in each mouse in the CD45.1/CD45.2 parabiotic mouse pairs. Green columns and white columns represent the percentages of CD45.1^+^ cells and CD45.2^+^ cells in splenic or hepatic pre-γδ T cells, respectively (*n* = 4). All results are presented as the mean ± SEM; *P < 0.05, **P < 0.01, ***P < 0.001 as determined by unpaired Student’s *t*-test for two-group comparisons

CD24 and CD73 are commonly regarded as surface markers that can be used to identify the developmental stages of γδ T cells.^20,21^ The developmental order associated with successive stages of γδ T cells is CD24^hi^CD73^−^ (progenitor cells) → CD24^+^CD73^+^ (precursor cells) → CD24^−^CD73^+^ (mature cells); alternatively, CD24^hi^CD73^−^ cells can develop directly into CD24^−^CD73^−^ cells. We compared the expression levels of *Cd24a* and *Nt5e* (CD73) among C1-T, C2-T, and C3-T/L based on data from the scRNA-seq analysis (Fig. 3a). The results showed that C1-T cells were associated with high expression levels of *CD24a* and almost no expression of *Nt5e*, which suggested that C1-T cells might be CD24^hi^CD73^−^ γδ T progenitor cells. C3-T/L had a low expression level of *Cd24a* and a high expression level of *Nt5e*, similar to CD24^+^CD73^+^ pre-γδ T cells. C2-T cells had moderate expression levels of *CD24a* and nearly no expression of *Nt5e*, indicating that C2-T cells might serve as a developmental transition phase between C1-T and C3-T/L.

To determine whether the murine liver contained pre-γδ T cells, we used flow cytometry to detect the protein expression levels of CD24 and CD73 on γδ T cells derived from the liver, thymus, spleen, and small intestine (Fig. 3b and Supplementary Fig. S3). We found that liver samples contained higher percentages of CD24^+^CD73^+^ pre-γδ T cells (15.7% ± 3.8%) than the spleen (6.4% ± 4.1%) and intestinal samples (1.0% ± 0.4%, Fig. 3c). The total number of pre-γδ T cells in the liver was comparable with that in the thymus (Fig. 3d), and the percentages of hepatic pre-γδ T cells increased with age (Fig. 3e). By analyzing the expression of CD27 and CD44, we found that the phenotype of liver pre-γδ T cells was CD27^−^CD44^hi/+^, whereas their counterparts in the thymus, spleen, and small intestine had a CD27^+^CD44^+/lo^ phenotype (Fig. 3f). We further detected the expression levels of the transcription factors T-cell-specific T-box transcription factor (T-bet) and retinoic acid-related orphan receptor gamma t (RORγt) in pre-γδ T cells. The results showed that hepatic pre-γδ T cells expressed significantly higher levels of T-bet and RORγt than those in the thymus or spleen (Fig. 3g). These results suggested that hepatic pre-γδ T cells were distinct from pre-γδ T cells in the thymus and spleen. To further evaluate the origins and properties of hepatic pre-γδ T cells, *CD45.1*^*+*^ and *CD45.2*^*+*^ B6 mice were joined by parabiosis, and the percentages of CD45.1^+^ and CD45.2^+^ pre-γδ T cells in the liver were measured two weeks later to evaluate the effects of chimerism. We found that liver CD24^+^CD73^+^ pre-γδ T cells expressing CD45.1 constituted nearly 90% of all pre-γδ T cells, with only 10% CD45.2^+^ pre-γδ T cells, whereas the opposite was observed in *CD45.2*^*+*^ parabiont mice (Fig. 3h), indicating that the majority of intrahepatic pre-γδ T cells were retained in the liver, although a small fraction of hepatic pre-γδ T cells were found in circulation. However, splenic pre-γδ T cells behaved in a circulating manner (Fig. 3h). These results demonstrated that the murine liver contains liver-resident pre-γδ T cells.

A previous study showed that CD44^lo^Ly-6C^−/+^ γδ T cells presented as naïve-like γδ T cells with high plasticity in vitro.^16^ To test whether liver CD24^+^CD73^+^ γδ T cells represent a group of naïve-like γδ T cells, the expression of CD44 and Ly-6C was analyzed on CD24^+^CD73^+^ γδ T cells from different tissues (Supplementary Fig. S4a). The results showed that the main phenotype of liver CD24^+^CD73^+^ γδ T cells was CD44^hi^Ly-6C^-/+^, which is inconsistent with the phenotype of naïve-like γδ T cells; in contrast, the phenotype of their counterparts in the thymus, spleen, and lymph node was CD44^lo^Ly-6C^−^ (Supplementary Fig. S4a). We then sorted CD24^+^CD73^+^ γδ T cells and assessed their plasticity to examine whether CD24^+^CD73^+^ γδ T cells could be polarized to produce different effector molecules if cultured under polarizing (Th1, Th17, and Treg-like) conditions. We found that hepatic CD24^+^CD73^+^ γδ T cells were unable to efficiently differentiate into either IFN-γ- or IL-17a-producing or Foxp3^+^ Treg cells, similar to thymic CD24^+^CD73^+^ γδ T cells (Supplementary Fig. S4b). These results suggested that hepatic CD24^+^CD73^+^ γδ T cells may not be a subset of naïve-like γδ T cells.

**Fig. 4:**
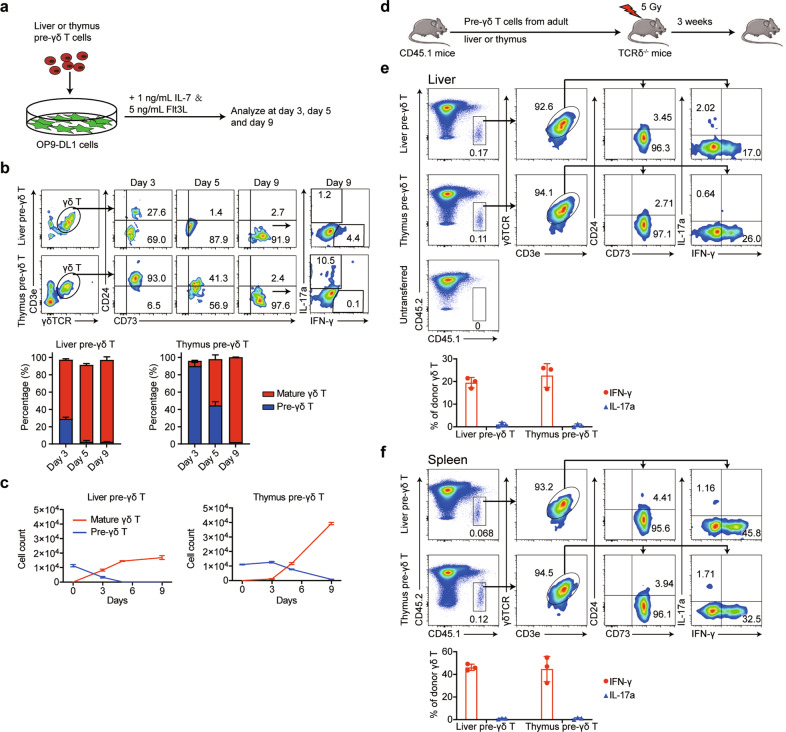
Liver-resident γδ T precursor cells can develop into mature γδ T cells, both in vitro and in vivo. **a** CD24^+^CD73^+^ γδ T cells sorted from the adult liver or thymus were cultured on OP9-DL1 monolayers in the presence of 1 ng/mL interleukin 7 (IL-7) with 5 ng/mL FMS-like tyrosine kinase 3 ligand (FLT3-L). **b** The percentages of pre-γδ T cells and mature γδ T cells were measured by analyzing the expression of CD24 and CD73 by flow cytometry on days 3, 5, and 9 (*n* = 3). The secretion levels of IFN-γ and IL-17a in CD24^−^CD73^+^ γδ T cells were detected by flow cytometry on day 9. **c** The absolute numbers of pre-γδ T cells and mature γδ T cells in the culture process (*n* = 3). **d** A schematic showing the experimental design. CD24^+^CD73^+^ γδ T cells (1 × 10^4^) sorted from the liver or thymus of *CD45.1*^*+*^ mice (6–8 weeks old) were intrasplenically transferred to 5 Gy-irradiated *TCRδ*^*−/−*^ mice (*CD45.2*^*+*^, 6–8 weeks old). **e**, **f** The expression of CD24 and CD73 and the secretion levels of IFN-γ and IL-17a were detected by flow cytometry gating on CD45.1^+^ donor-derived γδ T cells in the liver (**e**) or spleen (**f**) (*n* = 3)

Next, we investigated whether liver-resident pre-γδ T cells have the potential to differentiate into mature and functional γδ T cells. We sorted hepatic CD24^+^CD73^+^ pre-γδ T cells (cell purity can be observed in Supplementary Fig. S5) and cultured them on OP9-DL1 monolayers (Fig. 4a). On days 3, 5, and 9 of coculture, the developmental stages of γδ T cells were determined by analyzing the γδ T cell phenotypes by flow cytometry. On day 3, nearly 30% of the CD24^+^CD73^+^ pre-γδ T cells had become CD24^−^CD73^+^ γδ T cells, which increased to more than 90% at day 9 (Fig. 4b). These results indicated that hepatic pre-γδ T cells were able to differentiate into CD24^−^CD73^+^ mature γδ T cells in the in vitro coculture system. Similar results were observed from the coculture of thymus-derived pre-γδ T cells with OP9-DL1 monolayers (Fig. 4b). The ability to produce IFN-γ- and IL-17a-producing CD24^−^CD73^+^ γδ T cells was further detected by flow cytometry on day 9. The results showed that liver-derived pre-γδ T cells preferentially differentiated into IFN-γ-producing γδ T cells, with little differentiation into IL-17a-producing cells (Fig. 4b). In contrast, thymus-derived pre-γδ T cells only differentiated into IL-17a-producing γδ T cells (Fig. 4b). The total numbers of pre-γδ T cells and mature γδ T cells throughout the culture process were also counted (Fig. 4c).

We then isolated liver- or thymus-derived CD45.1^+^ pre-γδ T cells and adoptively transferred them into sublethally irradiated *CD45*.2^+^ *TCRδ*^*−/−*^ mice (Fig. 4d). After 3 weeks of re-establishment, we isolated CD45.1^+^ donor-derived γδ T cells and further analyzed the expression levels of CD24 and CD73. The results showed that transplanted liver-derived CD24^+^CD73^+^ pre-γδ T cells primarily differentiated into CD24^−^CD73^+^ mature γδ T cells in the liver, similar to the thymus-derived pre-γδ T cell transplantations (Fig. 4e, f). The intracellular cytokine secretion capacity was further analyzed, and γδ T cells that matured from either liver- or thymus-derived pre-γδ T cells were able to produce IFN-γ (Fig. 4e, f). These results indicated that liver-derived γδ T cell precursors could reside and develop in the liver. The liver microenvironment was suitable for the development of both liver-resident pre-γδ T cells and thymus-derived γδ T precursor cells. However, we also observed that transfusion of liver or thymus pre-γδ T cells was able to reconstruct γδ T cells in the spleen to some extent. These results implied that liver-derived pre-γδ T cells (which may also contain precursors that migrate from the thymus) could represent an alternative origin for intrahepatic and splenic γδ T cells, in addition to thymus-derived γδ T precursor cells.

### Liver hematopoietic progenitor LSM cells differentiate into IFN-γ^+^ γδ T cells

**Fig. 5:**
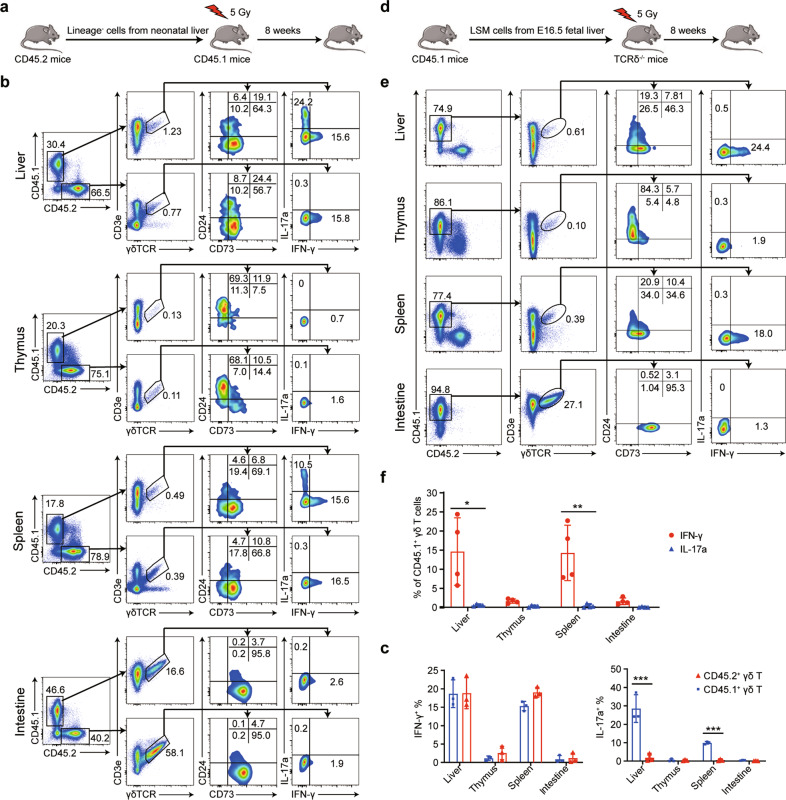
Fetal liver hematopoietic progenitor LSM cells differentiate into IFN-γ^+^ γδ T cells in the liver. **a** Schematic of the experimental design. Lineage^−^ cells (1 × 10^5^) sorted from the livers of neonatal *CD45.2*^*+*^ mice (B6) were intrasplenically transferred to 5 Gy-irradiated *CD45.1*^*+*^ mice (6 weeks old). **b** Flow cytometry was used to detect the expression of CD24 and CD73 to analyze the percentages of pre-γδ T cells and mature γδ T cells and the percentages of IFN-γ- and IL-17a-expressing cells among donor-derived and recipient γδ T cells after 8 weeks of transferring Lin^−^ cells from the neonatal mouse liver. **c** Statistical analysis of the percentages of IL-17a- and IFN-γ-expressing cells among CD45.1^+^ and CD45.2^+^ γδ T cells (*n* = 3). **d** Schematic of the experimental design. LSM cells (1 × 10^4^) sorted from E16.5 fetal livers of *CD45.1*^*+*^ mice were intrasplenically transferred to 5 Gy-irradiated *TCRδ*^*−/−*^ mice (*CD45.2*^*+*^, 6 weeks old). **e** Flow cytometry was used to analyze the percentages of pre-γδ T cells and mature γδ T cells and IFN-γ and IL-17a expression in CD45.1^+^ donor-derived γδ T cells 8 weeks after transferring LSM cells from E16.5 fetal livers. **f** Statistical analysis of the percentages of IL-17a- and IFN-γ-expressing cells among CD45.1^+^ γδ T cells (*n* = 4). All results are shown as the mean ± SEM; *P < 0.05, **P < 0.01, ***P < 0.001 as determined by unpaired Student’s *t*-test for two-group comparisons

The liver is the major hematopoietic organ during the fetal period, and HSPCs continue to reside in the liver after birth.^22,23^ Because the liver contains both HSPCs and liver-resident pre-γδ T cells, we wondered whether liver-resident pre-γδ T cells could be derived from HSPCs in the liver. We first sorted liver Lin^−^ cells from neonatal donor *CD45.2*^*+*^ mice (cell purity is shown in Supplementary Fig. S6a) and adoptively transferred the sorted cells to sublethally irradiated *CD45.1*^*+*^ recipient mice to evaluate the hematopoietic-reconstitution activity of γδ T cells (Fig. 5a). We analyzed the proportions of donor-derived mature and pre-γδ T cells in different tissues 8 weeks after transplantation. By gating CD45.1^+^ and CD45.2^+^ γδ T cells, no significant differences were observed in the percentages of donor-derived CD24^+^CD73^+^ pre-γδ T cells or CD24^−^CD73^+^ mature γδ T cells compared with those of the recipient-derived cells (Fig. 5b). Moreover, we found that liver γδ T cells reconstituted from transplanted Lin^−^ cells had the ability to produce IFN-γ but not IL-17a, in contrast to the results from the recipients (Fig. 5b, c). These results suggested that donor-derived liver HSPCs could differentiate into pre-γδ T cells and then into mature γδ T cells in recipient mice.

Previous studies have found that the fetal liver contains unique Lin^−^Sca-1^+^Mac-1^+^ hematopoietic stem and progenitor cells, which are phenotypically distinct from bone marrow-derived HSPCs.^24,25^ Although the majority of HPSCs migrate to the bone marrow after birth, where they remain until the adult stage, the liver retains LSM cells, which have hematopoietic reconstitution activity. We sorted LSM cells from the livers of embryonic day 16.5 (E16.5) *CD45.1*^*+*^ mice (cell purity is shown Supplementary Fig. S6b) and adoptively transferred 1 × 10^4^ LSM cells to sublethally irradiated *CD45.2*^*+*^ *TCRδ*^*−/−*^ recipient mice to determine whether LSM cells could differentiate into pre-γδ T cells and then into mature γδ T cells in the livers of the recipient animals (Fig. 5d). We found that the donor-derived CD24^+^CD73^+^ pre-γδ T cells were observed in the liver (Fig. 5e), and IFN-γ^+^ but not IL-17a^+^ γδ T cells could be detected in the tissues of the *CD45.2*^*+*^ *TCRδ*^*−/−*^ recipient mice (Fig. 5e, f), consistent with the results of Lin^−^ cell transplantation (Fig. 5b, c). These data indicated that fetal liver LSM cells were able to differentiate into IFN-γ^+^ γδ T cells. Similar results were obtained when transferring LSM cells sorted from the livers of neonatal mice (Supplementary Fig. S6c–e).

**Fig. 6:**
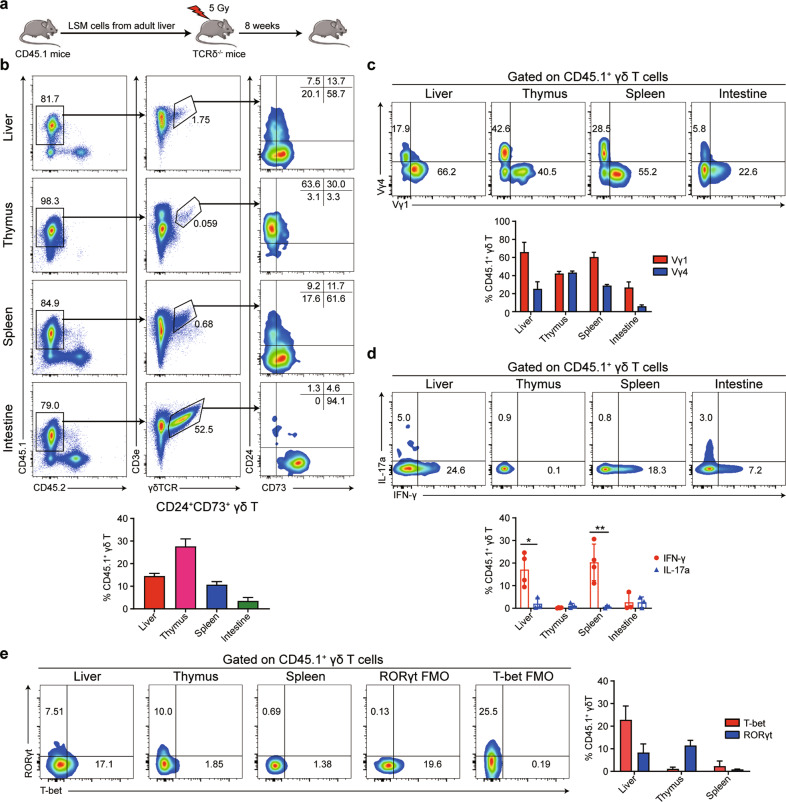
Adult liver LSM cells retain the developmental potential to differentiate into liver-resident IFN-γ^+^ γδ T cells in the liver. **a** Schematic of the experimental design. LSM cells (1 × 10^4^) sorted from the liver of *CD45.1*^*+*^ mice (6 weeks old) were intrasplenically transferred to 5 Gy-irradiated *TCRδ*^*−/−*^ mice (6 weeks old). **b**, **c** Flow cytometry was used to detect the percentages of CD45.1^+^ donor-derived pre-γδ T cells through the detection of CD24 and CD73 (**b**) and Vγ1 or Vγ4 chains on the CD45.1^+^ donor-derived γδ T cells (**c**, *n* = 3). **d**, **e** Flow cytometry was used to detect the intracellular levels of IFN-γ and IL-17a (**d**) and the expression levels of T-bet and RORγt (**e**) in the CD45.1^+^ donor-derived γδ T cells. Statistical analysis of the percentages of IL-17a and IFN-γ in the CD45.1^+^ γδ T cells (*n* = 3–4). All results are shown as the mean ± SEM; *P < 0.05, **P < 0.01 as determined by unpaired Student’s *t*-test for two-group comparisons

We further explored whether adult liver-derived LSM cells retained the capacity to differentiate into γδ T cells. A total of 1 × 10^4^ LSM cells obtained from the adult *CD45.1*^*+*^ mouse livers (cell purity is shown in Supplementary Fig. S7) were intrasplenically injected into sublethally irradiated *CD45.2*^*+*^ *TCRδ*^*−/−*^ recipient animals (Fig. 6a). The expression levels of CD24 and CD73 and the detection of Vγ1 or Vγ4 chains (the mouse Vγ gene nomenclature defined by Heilig and Tonegawa^26^) on donor-derived CD45.1^+^ γδ T cells were analyzed. As shown in Fig. 6b, c, donor-derived adult liver LSM cells were able to develop into pre-γδ T cells and mature γδ T cells based on the detection of Vγ1 and Vγ4 chains, with the comparative predominance of the Vγ1 chain in both the liver and spleen. Donor-derived γδ T cells were further analyzed to determine their capacity to secrete IFN-γ and IL-17a and for the expression of the transcription factors T-bet and RORγt. Donor-derived γδ T cells were able to secrete IFN-γ but secreted little IL-17a (Fig. 6d), consistent with the results obtained for fetal liver LSM cells (Fig. 5e, f). Donor hepatic γδ T cells expressed higher levels of T-bet than those in the thymus and spleen and similar levels of RORγt as those in the thymus (Fig. 6e). These data revealed that adult liver LSM cells retained the potential to develop into IFN-γ^+^ γδ T cells in the liver.

### IFN-γ-producing γδ T cells can originate from fetal liver LSM cells in a thymus-independent manner

**Fig. 7:**
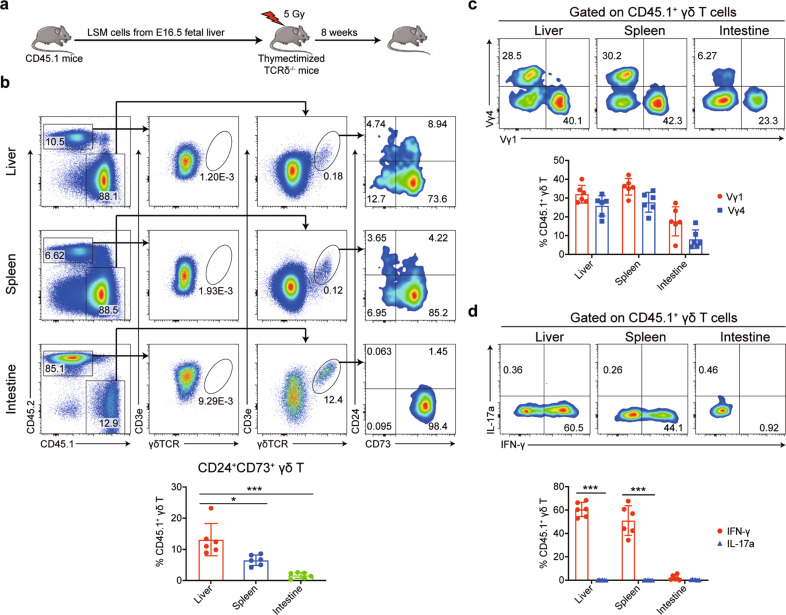
IFN-γ-producing γδ T cells can originate from fetal liver LSM cells independent of the thymus. **a** Schematic of the experimental design. Thymectomies were performed on 5–6-week-old *TCRδ*^*−/−*^ mice. Next, LSM cells (3 × 10^4^) were sorted from the E16.5 fetal liver tissue of *CD45.1*^*+*^ mice and intravenously transferred to 5 Gy-irradiated thymectomized *TCRδ*^*−/−*^ mice. **b** The negative control of γδ T cell gating was shown by gating CD45.2^+^ cells in recipient *TCRδ*^*−/−*^ mice. The percentages of donor-derived CD24^+^CD73^+^ pre-γδ T cells and CD24^−^CD73^+^ mature γδ T cells were analyzed by gating CD45.1^+^CD3e^+^γδTCR^+^ cells (*n* = 6). **c**, **d** Donor-derived CD45.1^+^ γδ T cells were further analyzed for the presence of Vγ1 or Vγ4 chains (**c**, *n* = 6) and the expression levels of IFN-γ and IL-17a (**d**, *n* = 6). All results are presented as the mean ± SEM; *P < 0.05, **P < 0.01, ***P < 0.001 as determined by unpaired Student’s *t*-test for two-group comparisons or one-way ANOVA for three group comparisons

To exclude the “conventional” thymus-dependent developmental pathway, particularly the potential that cells migrate from the thymus to the liver, we transferred fetal liver LSM cells into thymectomized *TCRδ*^*−/−*^ mice and evaluated the γδ T cell reconstitution activity associated with donor LSM cells (Fig. 7a). As shown in Fig. 7b, we gated CD45.2^+^ cells in the recipient *TCRδ*^*−/−*^ mice as a negative control for γδ T cell gating and then analyzed the percentages of donor-derived CD24^+^CD73^+^ pre-γδ T cells and CD24^−^CD73^+^ mature γδ T cells by gating CD45.1^+^ γδ T cells. The results showed that LSM cells derived from the fetal liver successfully reconstituted γδ T cells (Fig. 7b). The percentage of donor-derived pre-γδ T cells in the liver was higher than those in the spleen and intestine (Fig. 7b). Moreover, donor-derived γδ T cells in the liver and spleen primarily used the Vγ1 and Vγ4 chains, whereas γδ T cells in the intestine had a low level of the Vγ4 chain (Fig. 7c). Hepatic and splenic γδ T cells reconstituted in thymectomized mice had the potent capacity to produce IFN-γ (Fig. 7d). These data showed that fetal liver LSM cells could differentiate into functional γδ T cells in a thymus-independent manner. A similar conclusion was also obtained by transferring liver Lin^−^ cells from neonatal *CD45.1*^*+*^ mice to thymectomized *CD45.2*^*+*^ mice (Supplementary Fig. S8).

### Precursor cells from the fetal thymus develop into liver IL-17a^+^ γδ T cells

**Fig. 8:**
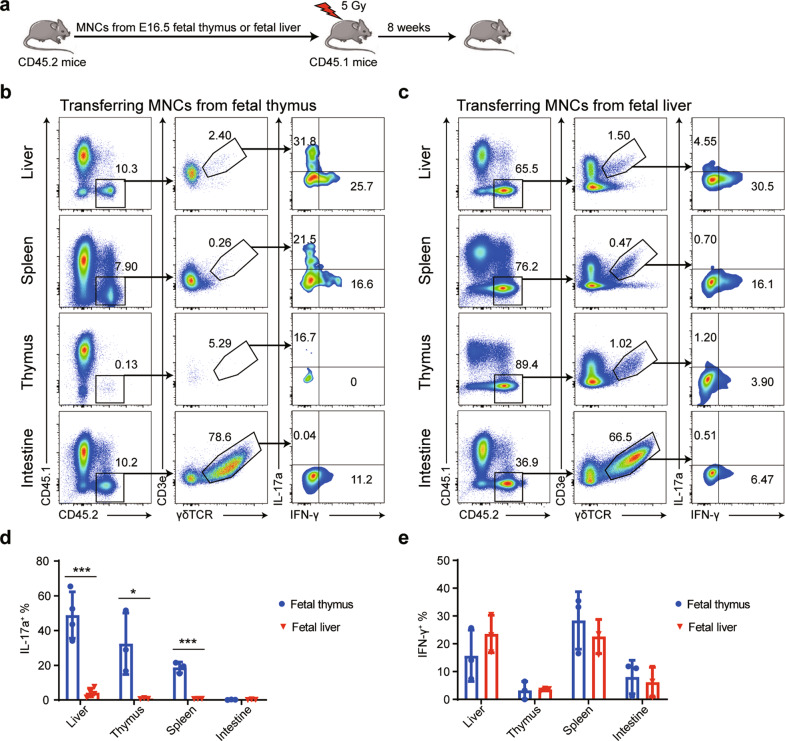
Progenitors from the fetal thymus but not the fetal liver produce liver IL-17a^+^ γδ T cells. **a** Schematic showing the experimental design. MNCs from E16.5 fetal thymus or liver (1×10^6^) tissue of *CD45.2*^*+*^ mice were intravenously transferred to 5 Gy-irradiated *CD45.1*^*+*^ mice (6–8 weeks old). **b**, **c** The proportions of CD45.1^+^ and CD45.2^+^ γδ T cells and the proportions of IFN-γ- and IL-17a-expressing CD45.1^+^ γδ T cells were detected by flow cytometry 8 weeks after cell transfer from the fetal thymus (**b**) or liver (**c**). **d**, **e** Statistical analysis of the expression of IL-17a (**d**) and IFN-γ (**e**) in the CD45.2^+^ γδ T cells in (**b**, **c**, *n* = 3–4). All results are presented as the mean ± SEM; *P < 0.05, **P < 0.01, ***P < 0.001 as determined by unpaired Student’s *t*-test for two-group comparisons

IL-17a^+^ γδ T cells have been shown to originate from early thymic progenitor cells and develop only in the fetal and perinatal thymus during a functional embryonic wave.^27^ We observed that the γδ T cells reconstituted from LSM cells (which only contain liver-specific HSPCs) derived from fetal, neonatal, or adult mice are able to secrete IFN-γ but not IL-17 (Figs. 5 and 6). These findings led us to explore whether liver-specific progenitors can only give rise to IFN-γ^+^ γδ T cells, whereas IL-17a^+^ γδ T cells originate from precursors in the thymus. We intravenously injected MNCs from the fetal liver or thymus into sublethally irradiated recipients to determine whether this was the case (Fig. 8a). We further measured the percentages of donor-derived IFN-γ^+^ γδ T and IL-17a^+^ γδ T cells after eight weeks. The results showed that the proportions of liver IL-17a^+^ γδ T cells in the recipient mice receiving fetal thymus MNC transplantations were 56.3% ± 11.2%, whereas only 3.43% ± 1.52% in the recipients receiving fetal liver MNC transplantations (Fig. 8b–d). No significant difference in the percentages of IFN-γ^+^ γδ T cells was observed between the two groups (Fig. 8e). These results suggested that the fetal thymus but not the liver contains precursors that have the potential to develop into IL-17a^+^ γδ T cells.

## Discussion

**Fig. 9:**
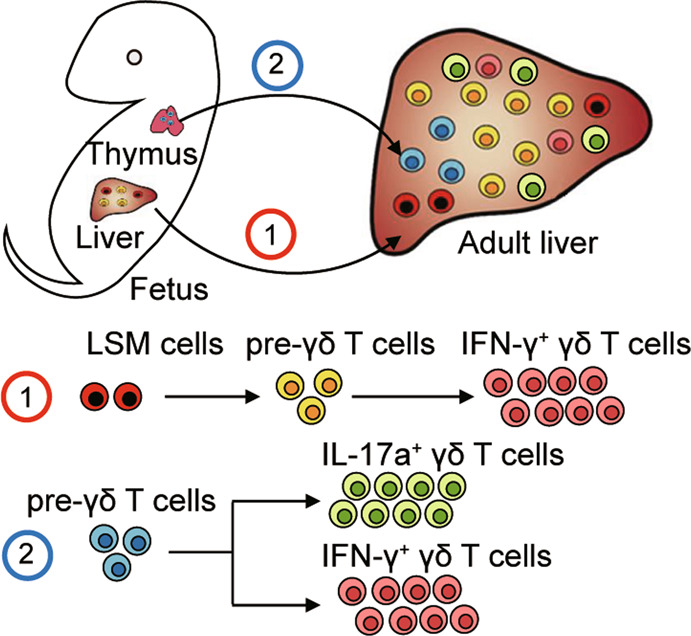
Schematic model showing the differentiation of liver LSM cells into pre-γδ T cells and IFN-γ^+^ γδ T cells in situ. Two origins exist for hepatic pre-γδ T cells and effector functional γδ T cells. ➀ γδ T cells develop in the fetal thymus and migrate to the periphery after birth. These γδ T cells can differentiate into IFN-γ^+^ and IL-17a^+^ γδ T cells. ➁ Fetal liver hematopoietic stem and progenitor LSM cells can differentiate into pre-γδ T cells and IFN-γ^+^ γδ T cells in situ, independent of the thymus. Importantly, adult liver LSM cells retain a similar developmental potential to differentiate into liver IFN-γ^+^ γδ T cells

γδ T cells are commonly believed to develop in the thymus, where CD4^−^CD8^−^ double-negative cells that receive strong TCR signaling predominantly develop into γδ T cells.^28^ Recently, increasing studies have focused on determining the developmental origins of γδ T cells, including the distinct developmental pathways and transcriptional programs associated with various γδ T subsets.^14,29–31^ To date, many questions regarding the differentiation of γδ T cells have remained unsolved, with contradictory findings and no consensus. The existence of an extrathymic developmental pathway for γδ T cells has been reported, as demonstrated in murine intestinal cryptopatches and Peyer’s patches.^10,32^ Increasing evidence has suggested that the liver contains HSPCs and liver-resident γδ T cells,^7,22^ although whether liver-resident γδ T cells could develop in the liver has not yet been explored. In the present study, we first analyzed the profiles and heterogeneity of γδ T cells, identifying eight distinct clusters, including clusters in the thymus that displayed features consistent with a developing population and a γδ T17-like cluster. The liver not only contained clusters with features similar to γδ T1, γδ T17, and γδ NKT subpopulations but also featured two unidentified clusters, C7 and C8. Notably, we identified an immature developing cluster (C3-L) in the liver, implying the existence of γδ T precursor cells in the liver. We further defined the developmental trajectory of γδ T cells based on pseudotime-based cell ordering analysis. Furthermore, we confirmed that the liver contains liver-resident pre-γδ T cells with the ability to develop into mature functional γδ T cells. Moreover, we verified that liver hematopoietic progenitor LSM cells could differentiate into pre-γδ T cells and mature γδ T cells in the liver. Importantly, our results showed that LSM cells were able to differentiate into IFN-γ^+^ γδ T cells but not into IL-17a^+^ γδ T cells, which was confirmed in thymectomized mouse models. These results suggested that IFN-γ-producing γδ T cells could originate from liver LSM cells independent of the thymus, whereas fetal thymus-derived progenitors could develop into both IFN-γ^+^ and IL-17a^+^ γδ T cells. We propose that liver pre-γδ T cells and mature γδ T cells originate from two distinct populations. One population of liver pre-γδ T cells originates from the thymus and migrates to the liver, where they further develop into IL-17a^+^ γδ T cells; another population of liver γδ T precursor cells differentiates from liver-specific LSM cells, primarily generating IFN-γ^+^ γδ T cells from the embryonic stage through adulthood (Fig. 9). To our knowledge, this is the first study to define the unique characteristics of intrahepatic γδ T cells with regard to heterogeneity, origins, and differentiation.

γδ T cells are a heterogeneous population consisting of multiple subsets. Based on cytokine production, γδ T cells are commonly divided into IFN-γ-producing γδ T cells (γδ T1) and IL-17-producing γδ T cells (γδ T17). Later, other subsets, such as γδ NKT, regulatory γδ T, and memory γδ T cells, were reported.^33–35^ Some γδ T cell subsets are predominantly located in particular tissues and exhibit specific phenotypes and tissue-resident features,^7,34,36^ demonstrating the phenotypic and functional plasticity of γδ T cells. The liver is a unique immunological organ enriched with γδ T cells that play crucial roles in the maintenance of liver homeostasis and defense against infection and cancer. Using scRNA-seq analysis, we identified 6 liver γδ T cell subsets. By comparing differences in gene expression, we distinguished C6-T/L as a population of γδ T17-like cells expressing *Il17a*, *Il17f*, and *Rorc*. C4-L, C5-L, and C7-L expressed γδ T1 cell signature genes, including *Ifng* and *Tbx21*. C5-L and C7-L were characterized by cytotoxic γδ T cell subsets with higher expression levels of *Gzma* and *Gzmb*, whereas C4-L had some features of γδ NKT cells.^37^ In addition, we found a small population of C8-L cells, enriched in the expression of *Apoe*, *Lyz2*, *Cd74*, *Ifitm2, Cxcl2, Cst3*, and *Lst1*, which is inconsistent with known subsets. *Lyz2* encodes lysozyme C-2, which has a bacteriolytic function, and *Ifitm2* encodes interferon-induced transmembrane protein 2, which inhibits viral entry into host cells. Thus, we speculate that C8-L may represent a novel γδ T cell subset that exerts antimicrobial and antiviral activity. However, due to the too low proportion of cells in this subset, we could not exclude the possibility that this cluster represents a doublet or technical artifact, requiring further verification. Collectively, the heterogeneity of liver γδ T cells suggests that hepatic γδ T cells play a variety of critical roles in immune defenses, immunosurveillance, and immune homeostasis.

Researchers have long attempted to determine the developmental and differentiation pathways associated with γδ T cells. Although several models have been proposed to explain the intrathymic γδ T cell commitment process, the underlying mechanistic differentiation programs have remained largely unclear. Based on our scRNA-seq analysis, we identified that the C1-T, C2-T, and C3-T in the thymus were likely developing cells representing the early stages of γδ T cell development. The pseudotime-based cell ordering analysis further facilitated the visualization of the developmental and differentiation trajectory: C1-T → C2-T → C3-T/L → C4/5/7-L or C6-T/L/C8-L. C1-T γδ T progenitor cells develop through the C2-T intermediate stage into C3-T/L γδ T precursor cells. C3-T/L precursor cells were positioned at the point of developmental divergence and were able to generate C4-L, C5-L, or C7-L subsets or give rise to C6-T/L or C8-L subpopulations. This defined developmental trajectory is consistent with related studies based on gene profiles and successive changes in gene expression.^13,14^ The high expression level of *Cd24a* and the lack of *Nt5e* expression in C1-T cells were consistent with the previously reported CD24^hi^CD73^−^ phenotype of γδ T progenitor cells.^20^ *Nt5e* encodes CD73, a TCR ligand-induced cell surface protein, and almost no *Nt5e* was detected in C1-T and C2-T, indicating that C2-T is located at the developmental stage before TCR selection. *Nt5e* is upregulated in C3-T/L, suggesting the acquisition of TCR signaling. From C1-T to C2-T, C3-T/L and C4-L, C5-L or C6-T/L, the expression level of *CD24a* decreased sequentially, consistent with the expression pattern (from CD24^high^ to CD24^low^) observed during the differentiation and developmental process of progenitor, precursor, immature, and mature γδ T cells.^13,28^ These results suggest that the pseudotemporal maturation trajectory is theoretically appropriate. However, the accuracy and reliability of the trajectory require further confirmation and may be readjusted with the collection of experimental data.

The fetal liver is the major hematopoietic organ. After birth, HPSCs migrate to and reside in the bone marrow. However, the adult liver continues to retains a small population of LSM cells, which have the capacity for hematopoietic reconstitution and can further differentiate into mature immune cells.^24,25^ γδ T cells primarily develop in the fetal thymus and migrate into the periphery after birth. Several studies have shown that γδ T cells may develop and differentiate outside of the thymus. A typical example is the intestinal mucosa, including cryptopatches and Peyer’s patches, where extrathymic γδ T (γδ IEL) development occurs depending on the secretion of the cytokine IL-7 and stem cell factor (SCF) from the epithelial cells of the small intestine.^32^ Similar to the thymus and small intestine, the liver microenvironment supports hematopoiesis and lymphocyte differentiation by secreting hematopoiesis-promoting cytokines, such as IL-7 and SCF.^12,38^ Whether liver-specific LSM cells can differentiate into intrahepatic γδ T cells in situ remains unknown. In the present study, based on the scRNA-seq analysis, we found that γδ T precursor cells (C3-T/L) exist not only in the thymus but also in the liver. The existence of pre-γδ T cells was confirmed by flow cytometric detection of CD24 and CD73 expression. We confirmed that both hepatic CD24^+^CD73^+^ pre-γδ T cells and liver-specific LSM cells could differentiate into mature functional γδ T cells. However, γδ T cells differentiated from LSM cells have the capacity to secrete IFN-γ but not IL-17a, whereas fetal thymus-derived progenitor cells primarily develop into IL-17a-producing γδ T cells. In a thymectomized mouse model, we further confirmed that hepatic pre-γδ T cells and IFN-γ-producing mature γδ T cells could develop from liver LSM cells in a thymus-independent manner. These results suggested that liver pre-γδ T cells and mature γδ T cells may originate from two populations. In the thymus, γδ T progenitor cells (C1-T) differentiate into the pre-γδ T cell stage (C3-T), some of which further differentiate into γδ T17 cells in the thymus and migrate to the periphery, whereas others migrate into and then reside in the liver, where they further develop into IL-17a^+^ γδ T cells. In the liver, another population of liver γδ T precursor cells primarily develops into IFN-γ^+^ γδ T cells and differentiates from liver-specific LSM cells from the embryonic stage through adulthood. Similarly, liver IL-17-producing γδ T cells and some populations of IFN-γ-producing γδ T cells may not share developmental origins. Liver IL-17-producing γδ T cells originate from the thymus, whereas a subpopulation of IFN-γ-producing γδ T cells likely originates from liver LSM cells, although others may also develop from the thymus. By comparing the phenotypes of the thymus and liver pre-γδ T cells, we observed distinct features associated with hepatic pre-γδ T cells. As shown in Fig. 3e, the thymus- and spleen-derived pre-γδ T cells had a CD27^+^CD44^+/lo^ phenotype, whereas the liver pre-γδ T cells were primarily CD27^−^CD44^hi/+^, indicating that most liver pre-γδ T cells might not migrate from the thymus, consistent with the results of the parabiosis model. Together, our findings provide clues to support two potential origins for hepatic pre-γδ T cells and indicate that the liver is a critical site for the extrathymic development of γδ T cells, although the exact developmental mechanism and program remain to be further clarified.

IL-17a^+^ γδ T cells have long been known to develop exclusively in the embryonic or perinatal thymus through a pre-programmed effector fate, and these cells persist in adult mice as self-renewing, long-lived cells.^27,29,39,40^ Our results showed that precursors from the fetal thymus develop into IL-17a^+^ γδ T cells, consistent with previous studies. These findings support the view that IL-17a-producing γδ T cells do not share developmental origins with IFN-γ-producing γδ T cells. We propose that liver IL-17-producing γδ T cells originate from the fetal thymus, whereas at least some subpopulations of IFN-γ-producing γδ T cells originate from liver LSM cells, although others develop from the thymus. Thus, we revised the pseudotemporal ordering of γδ T cells that differentiate from progenitor cells in the fetal thymus as follows: C1-T → C2-T → C3-T/L → C4/5/7-L and C6-T/L. Several recent studies have proposed that γδ T17 cells may have distinct progenitors from other γδ T cell subsets (γδ T1 and γδ NKT cells). γδ T17 progenitor cells may originate before the expression of TCRγδ and thus have different developmental potentials.^13,14,29^ Whether liver IL-17-producing γδ T cells can develop from TCRγδ-negative progenitors requires further investigation. The question of why LSM-derived γδ T cells have different features and predominantly produce IFN-γ but not IL-17a requires further investigation. We postulate that such differences might stem from both intrinsic and extrinsic factors. On the one hand, distinct properties that distinguish LSM and thymus-derived progenitor or precursor cells might determine their different developmental fates. Figure 2d shows the differentially expressed genes between these cell types, including genes encoding transcription factors, key molecules, and receptors, all of which might contribute to different fates for γδ T cell development and differentiation. On the other hand, differences in the liver and thymus microenvironments may provide different signals, which may be necessary for progenitor cell acquisition of cytokine-secreting effector fates.

In summary, our study comprehensively deciphered and identified distinct hepatic γδ T cell subsets and their characteristics. We revealed the developmental trajectory of γδ T cells. More importantly, we provided evidence that the liver is a site of extrathymic γδ T cell development. Liver LSM-derived γδ T cells differentiate through distinct developmental pathways and cytokine-secreting effector fates compared with γδ T cells that originate from the thymus. More evidence is needed to better dissect the developmental program and related mechanisms. The relative contributions of liver-derived and thymus-derived precursors to the seeding of the liver under normal circumstances must be determined. However, currently available experimental techniques and tools may not be equipped for this level of detail. In vivo fate mapping systems may provide more definitive and physiologically relevant insights into the behavioral dynamics and footprints of cellular differentiation pathways associated with stem and progenitor cells in vivo.^41^ Unfortunately, no specific gene or marker has yet been identified that can be used to trace liver-derived LSM cells. Further determination of the functional divisions and contributions (such as maintaining immune tolerance or exerting immune surveillance) between liver- and thymus-derived γδ T cell populations in normal and disease conditions should be explored. However, the present study demonstrated that examining the heterogeneity and unique developmental features of hepatic γδ T cells could offer insight into their critical roles in diverse immune effector functions and liver-related diseases.

## Supplementary information

Supplementary Tables and Figures
