# Supplementary material for: Single-cell analysis reveals the origins and intrahepatic development of liver-resident IFN-γ-producing γδ T cells
Source: Cell Mol Immunol. 2021 Mar 10;18(4):954–68. doi: 10.1038/s41423-021-00656-1 (PMC8115257; doi:10.1038/s41423-021-00656-1)
Supplement: Supplementary file 1 — Supplementary Tables and Figures [file 41423_2021_656_MOESM1_ESM.docx]

**Single-cell analysis reveals the origins and intrahepatic development of liver-resident IFN-γ-producing γδ T cells**

Yuan Hu^1^, Keke Fang^1^, Yanan Wang^1^, Nan Lu^2^, Haoyu Sun^3^, Cai Zhang^1^

^1^Institute of Immunopharmaceutical Sciences, School of Pharmaceutical Sciences, Cheeloo College of Medicine, Shandong University, Jinan, Shandong 250012, China

^2^Institute of Diagnostics, School of Medicine, Cheeloo College of Medicine, Shandong University, Jinan, Shandong 250012, China

^3^Institute of Immunology, School of Basic Medical Sciences, Division of Life Sciences and Medicine, University of Science and Technology of China, Hefei, Anhui 230027, China

**Supplemental Table 1. Antibody information**

| Antibodies |  |  |
| --- | --- | --- |
| PE/CF594 anti-mouse CD45.1 antibody | BD | Cat#562452 |
| PerCP/Cy5.5 anti-mouse CD3e antibody | eBioscience | Cat#45-0031-82 |
| APC/eFlu780 CD24 antibody | eBioscience | Cat#47-0242-82 |
| PE CD27 antibody | eBioscience | Cat#12-0271-82 |
| eFlu450 anti-mouse CD73 antibody | eBioscience | Cat#48-0731-82 |
| PerCP/eFlu710 anti-mouse CD73 antibody | eBioscience | Cat#46-0731-80 |
| PE/Cy7 anti-human/mouse T-bet antibody | eBioscience | Cat#25-5825-82 |
| PE/eFlu610 anti-mouse RORγt antibody | eBioscience | Cat#61-6981-82 |
| FITC anti-mouse Ly-6G/Ly-6C (Gr-1) antibody | eBioscience | Cat#11-5931-82 |
| PE/Cy7 anti-mouse Ly-6C antibody | eBioscience | Cat#25-5932-80 |
| PE anti-mouse FOXP3 antibody | eBioscience | Cat#12-5773-80 |
| Fixable Viability Dye eFluor 506 | eBioscience | Cat#65-0863-18 |
| FITC anti-mouse CD24 antibody | Biolegend | Cat#101806 |
| Fluor 700 an Alexa ti-mouse CD45.2 antibody | Biolegend | Cat#109822 |
| PE/Cy7 anti-mouse CD3 antibody | Biolegend | Cat#100220 |
| PerCP/Cy5.5 anti-mouse CD3e antibody | Biolegend | Cat#100328 |
| FITC anti-mouse CD19 antibody | Biolegend | Cat#115506 |
| FITC anti-mouse/human CD44 antibody | Biolegend | Cat#103006 |
| APC anti-mouse CD45.2 antibody | Biolegend | Cat#109814 |
| APC anti-mouse TCRγ/δ antibody | Biolegend | Cat#118116 |
| PE/Cy7 anti-mouse TCR γ/δ antibody | Biolegend | Cat#118124 |
| APC anti-mouse TCR Vγ1/Cr4 antibody | Biolegend | Cat#141107 |
| FITC anti-mouse TCR Vγ4 antibody | Biolegend | Cat#137704 |
| APC anti-mouse Ly-6A/E (Sca-1) antibody | Biolegend | Cat#108112 |
| PE anti-mouse/human CD11b antibody | Biolegend | Cat#101208 |
| FITC anti-mouse CD11c antibody | Biolegend | Cat#117306 |
| FITC anti-mouse NK1.1 antibody | Biolegend | Cat#108705 |
| FITC anti-mouse Ter119 antibody | Biolegend | Cat#116205 |
| PE anti-mouse IFN-γ antibody | Biolegend | Cat#505808 |
| PE/Dazzle 594 anti-mouse IFN-γ antibody | Biolegend | Cat#505845 |
| BV421 anti-mouse IL-17a antibody | Biolegend | Cat#506926 |
| PE/Dazzle 594 anti-mouse IL-17a antibody | Biolegend | Cat#506938 |

**Supplemental Table 2. The cell counts and purity of clusters acquired from single-cell RNA sequencing**

|  | **C1** | **C2** | **C3** | **C4** | **C5** | **C6** | **C7** | **C8** | **Total** |
| --- | --- | --- | --- | --- | --- | --- | --- | --- | --- |
| Thymus | 1081  (60.5%) | 405  (22.7%) | 173  (9.68%) | 4  (0.22%) | 15  (0.84%) | 105  (5.88%) | 2  (0.11%) | 2  (0.11%) | 1787  (100%) |
| Liver | 0  (0%) | 19  (0.33%) | 542  (9.36%) | 1839 (31.8%) | 2656  (45.8%) | 455  (7.86%) | 177  (3.06%) | 102  (1.76%) | 5790  (100%) |

**Supplemental Figures**

**
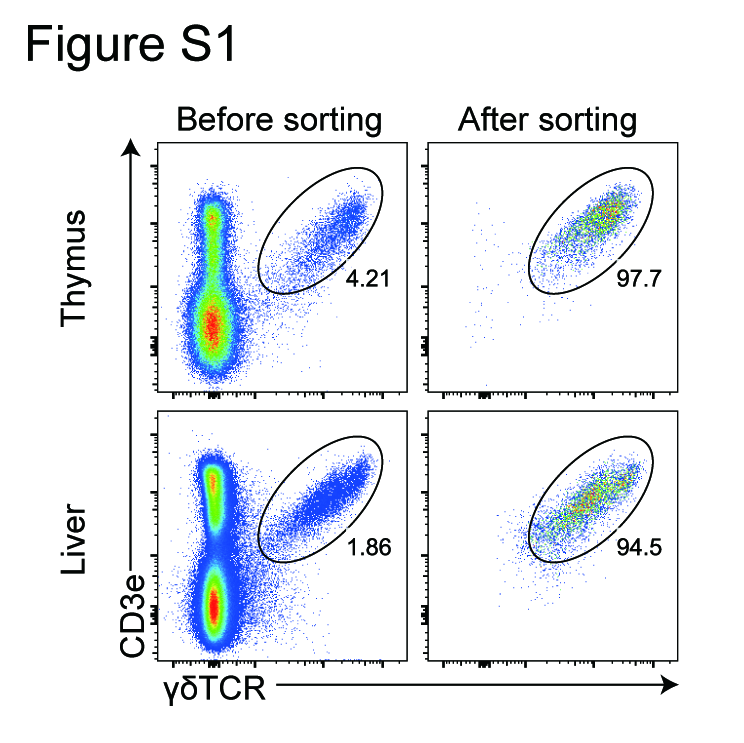
**

**Supplemental Figure 1. The purity of sorted γδ T cells.** γδ T cells were sorted from thymus and liver tissue for single-cell RNA sequencing. The purity was 97.7% for thymic γδ T cells and 94.5% for hepatic γδ T cells.

**
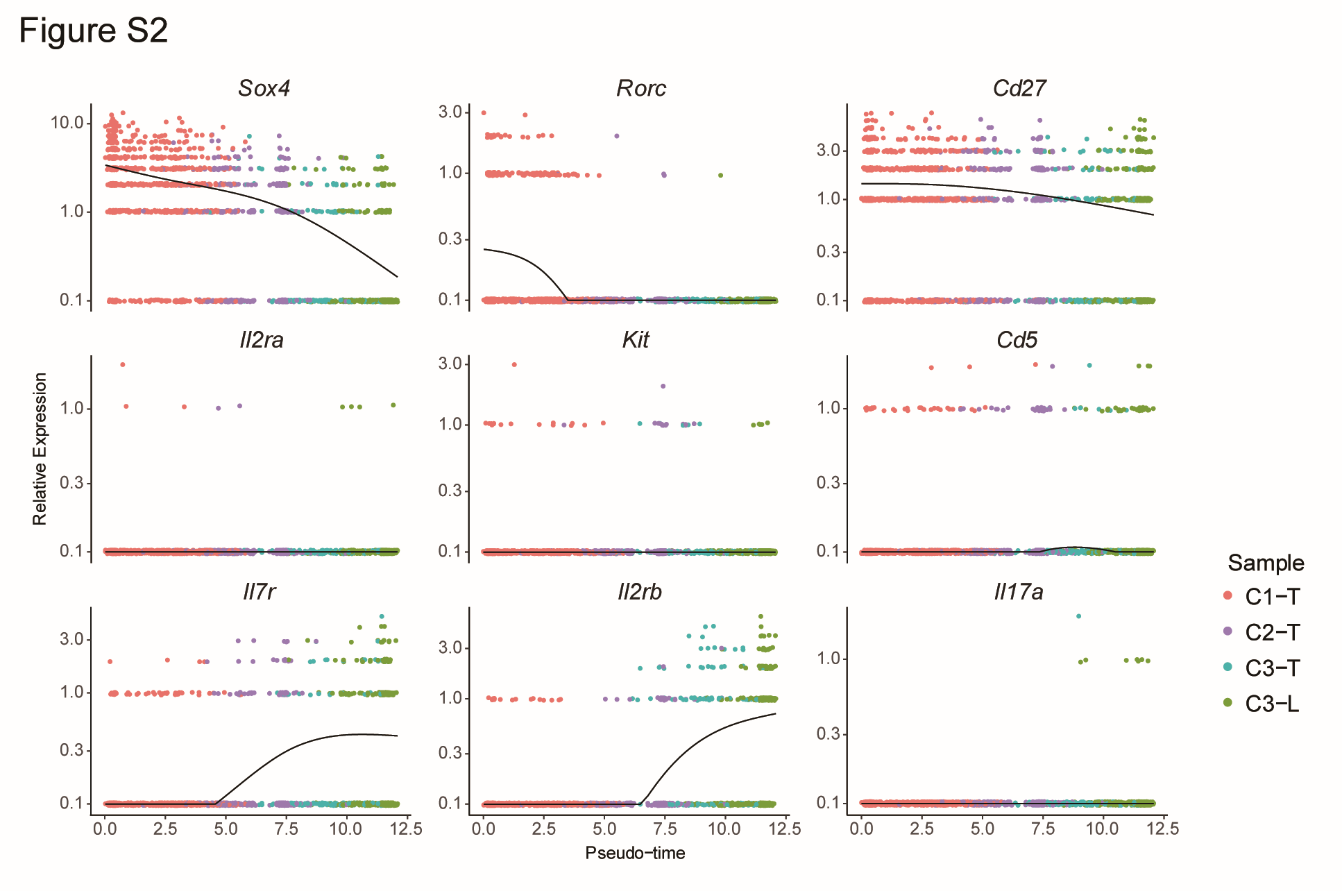
**

**Supplemental Figure 2. The expression trajectories of selected genes were analyzed by pseudotemporal ordering.** The expression trajectories of selected genes, including *Sox4*, *Rorc*, *Cd27*, *Il2ra*, *Kit*, Cd5, *Il7r*, *Il2rb,* and *Il17a*, were analyzed by pseudotemporal ordering among C1-T, C2-T, C3-T, and C3-L.

**
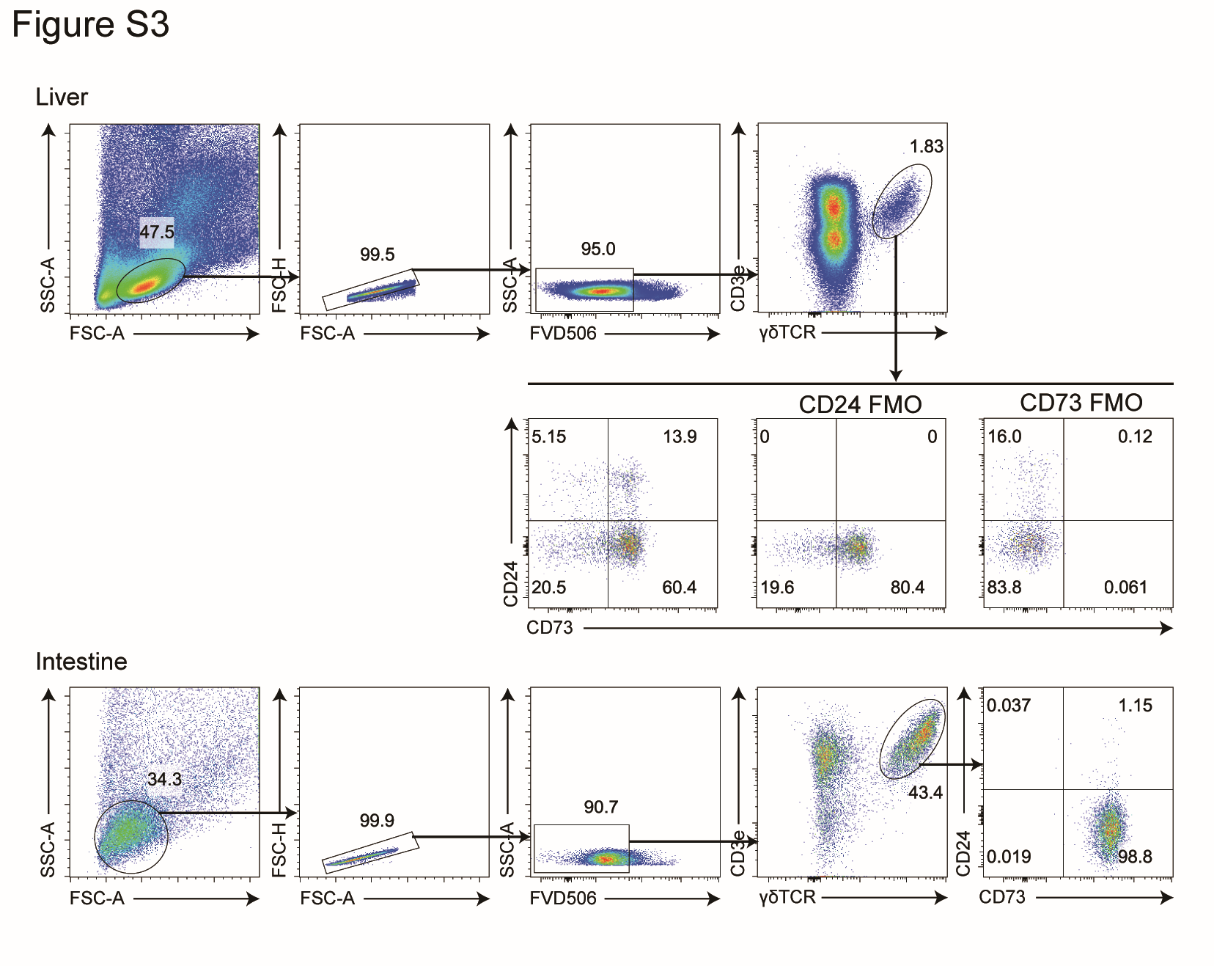
**

**Supplemental Figure S3. The gating strategy for liver and intestinal γδ T precursor cells.** First, we gated lymphocytes. Second, single-cells were gated through FSC-A and FSC-H. Dead cells were excluded by FVD 506 staining. Next, we gated CD3e^+^ and γδTCR^+^ cells as γδ T cells to further analyze the expression of CD24 and CD73. CD24 and CD73 FMO controls were used to confirming the gating sites. We adjusted the CD3e^+^γδTCR^+^ gating position for intestinal cells due to different expression patterns of CD3e and γδTCR compared with liver, spleen, and thymus cells.

**
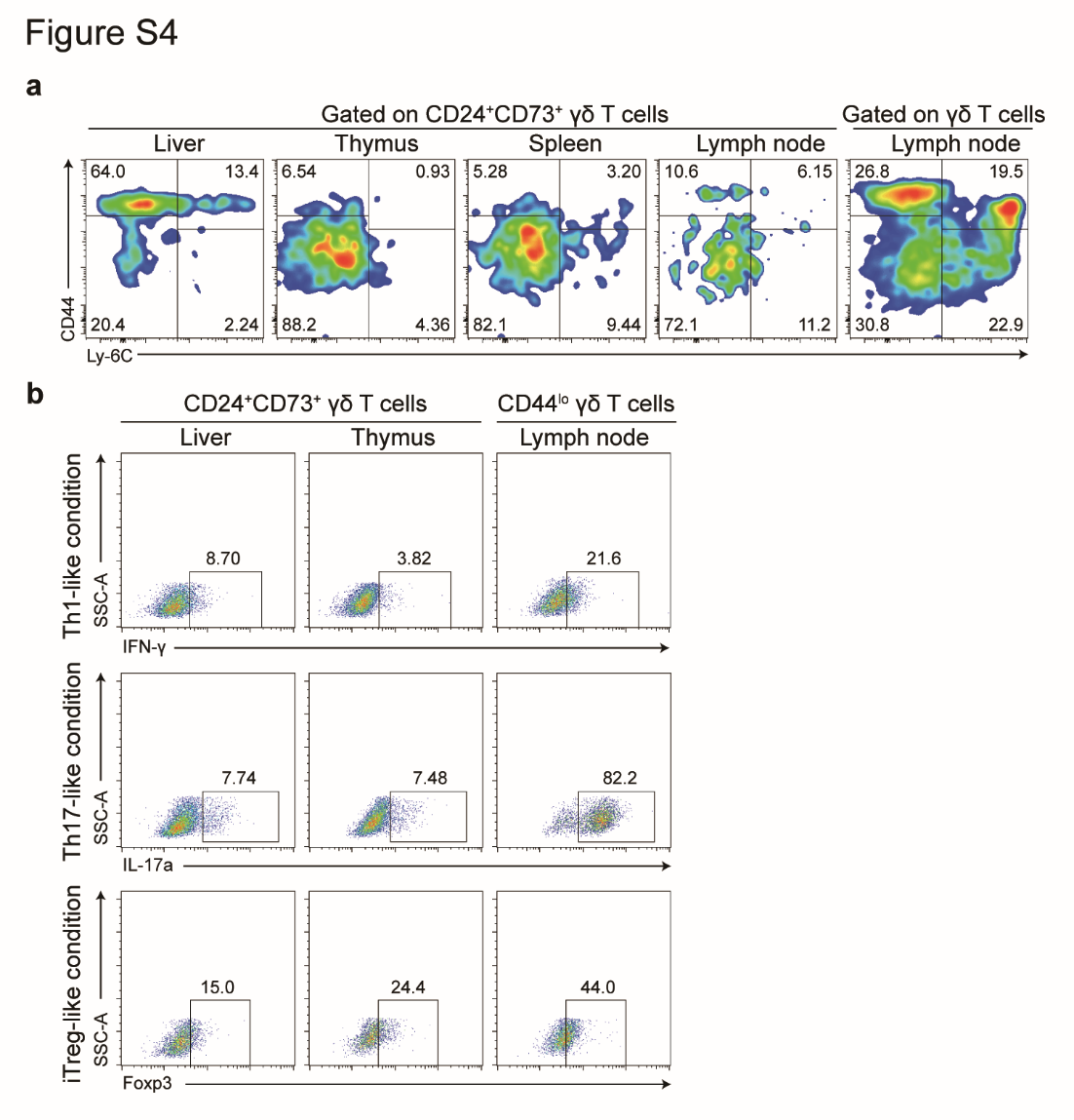
**

**Supplemental Figure S4. Hepatic CD24^+^CD73^+^ γδ T cells are not a subset of naïve-like γδ T cells. a** Flow cytometry was applied to analyze the expression of CD44 and Ly-6C on CD24^+^CD73^+^ γδ T cells from the liver, thymus, spleen, and lymph node. **b** To assess the plasticity of pre-γδ T cells, *in vitro* polarization assays were performed. CD24^+^CD73^+^ pre-γδ T cells from the thymus and liver as well as CD44^lo^ naïve γδ T cells from the lymph node were sorted by flow cytometry and then stimulated for 4 days with coated anti-CD3 (clone 145.2C11) and anti-CD28 (clone 37.51) antibodies, both at 4 μg/mL, in the presence of human IL-2 (13 ng/mL) and mouse IL-12 (10 ng/mL) to create Th1-like conditions; in the presence of anti-IFN-γ (clone R4-6A2) neutralizing antibody (5 μg/mL), mouse IL-6 (20 ng/mL), mouse IL-23 (10 ng/mL), and human TGF-β1 (10 ng/mL) to create Th17-like conditions; and in the presence of human IL-2 (13 ng/mL) and human TGF-β1 (10 ng/mL) to create iTreg-like conditions. The expression levels of IFN-γ, IL-17a, and Foxp3 were measured.

**
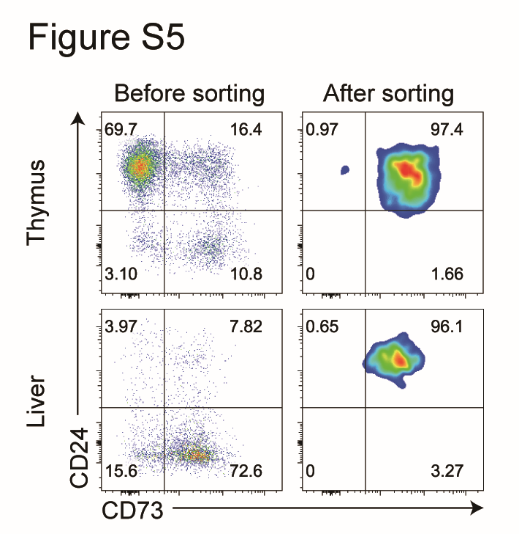
**

**Supplemental Figure 5. The purity of sorted CD24^+^CD73^+^ γδ T cells.** The purity of CD24^+^CD73^+^ γδ T cells sorted from the thymus or liver was 97.4% or 96.1%, respectively.

**
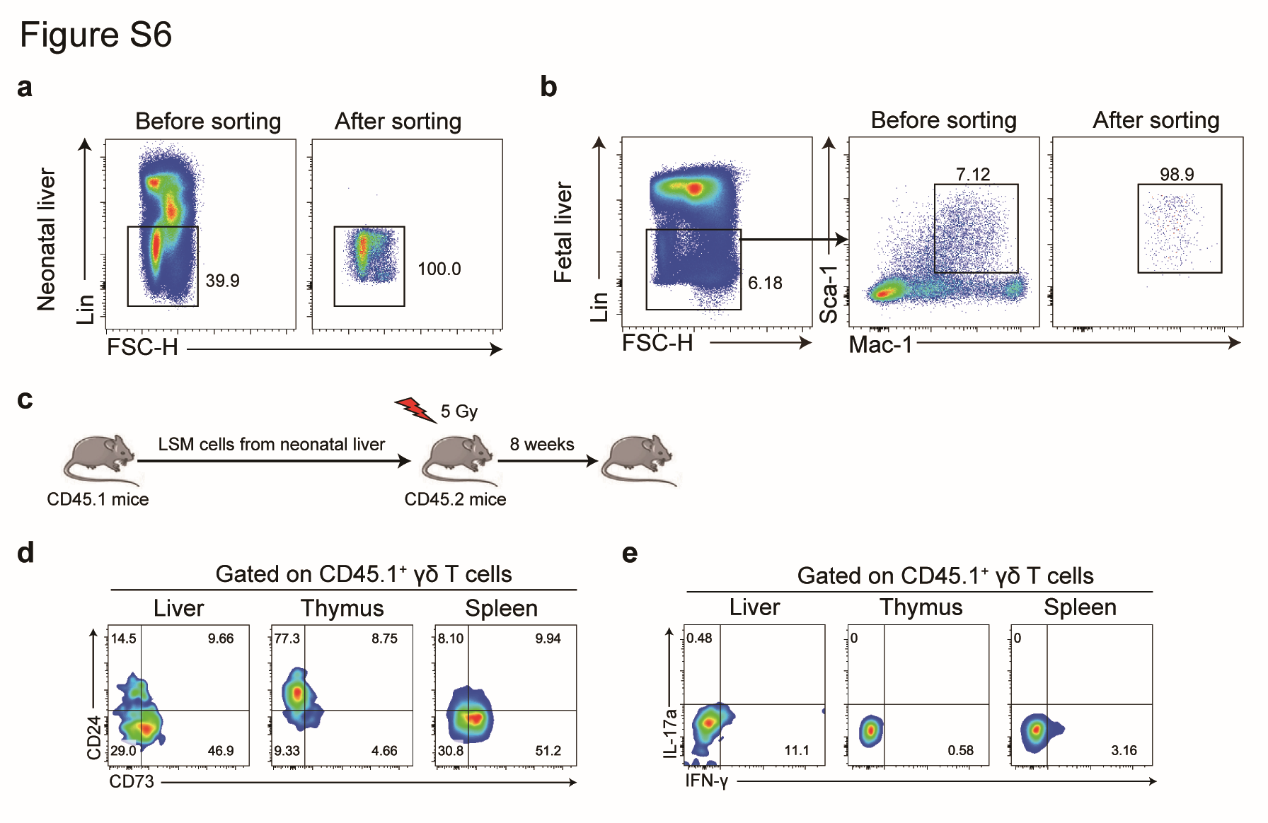
**

**Supplemental Figure S6. Neonatal liver hematopoietic progenitor LSM cells differentiate into IFN-γ^+^ γδ T cells. a** The purity of sorted neonatal liver lin^−^ cells was 100%. **b** The purity of sorted E16.5 fetal liver LSM cells was 98.9%. **c** Schematic of the experimental design. LSM cells (1 × 10^4^) sorted from neonatal fetal liver tissue from *CD45.1^+^* mice were intrasplenically transferred into 5 Gy-irradiated *CD45.2^+^* mice. **d** and **e** Flow cytometry was used to analyze the percentages of pre-γδ T cells and mature γδ T cells (**d**), IFN-γ and IL-17a (**e**) in CD45.1^+^ donor-derived γδ T cells 8 weeks after cell transfer.

**
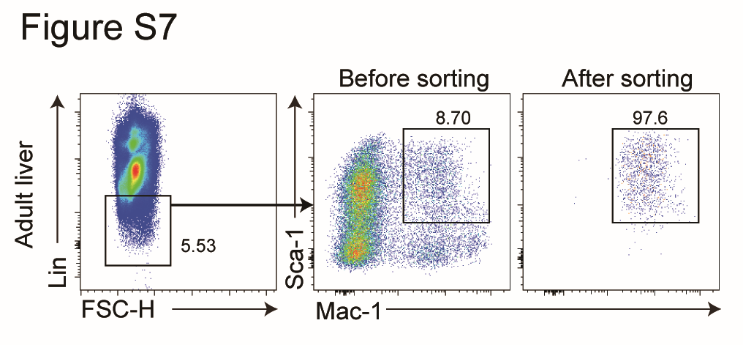
**

**Supplemental Figure 7. The purity of sorted adult liver LSM** **cells.** The purity of sorted adult liver LSM cells was 97.6%.

**
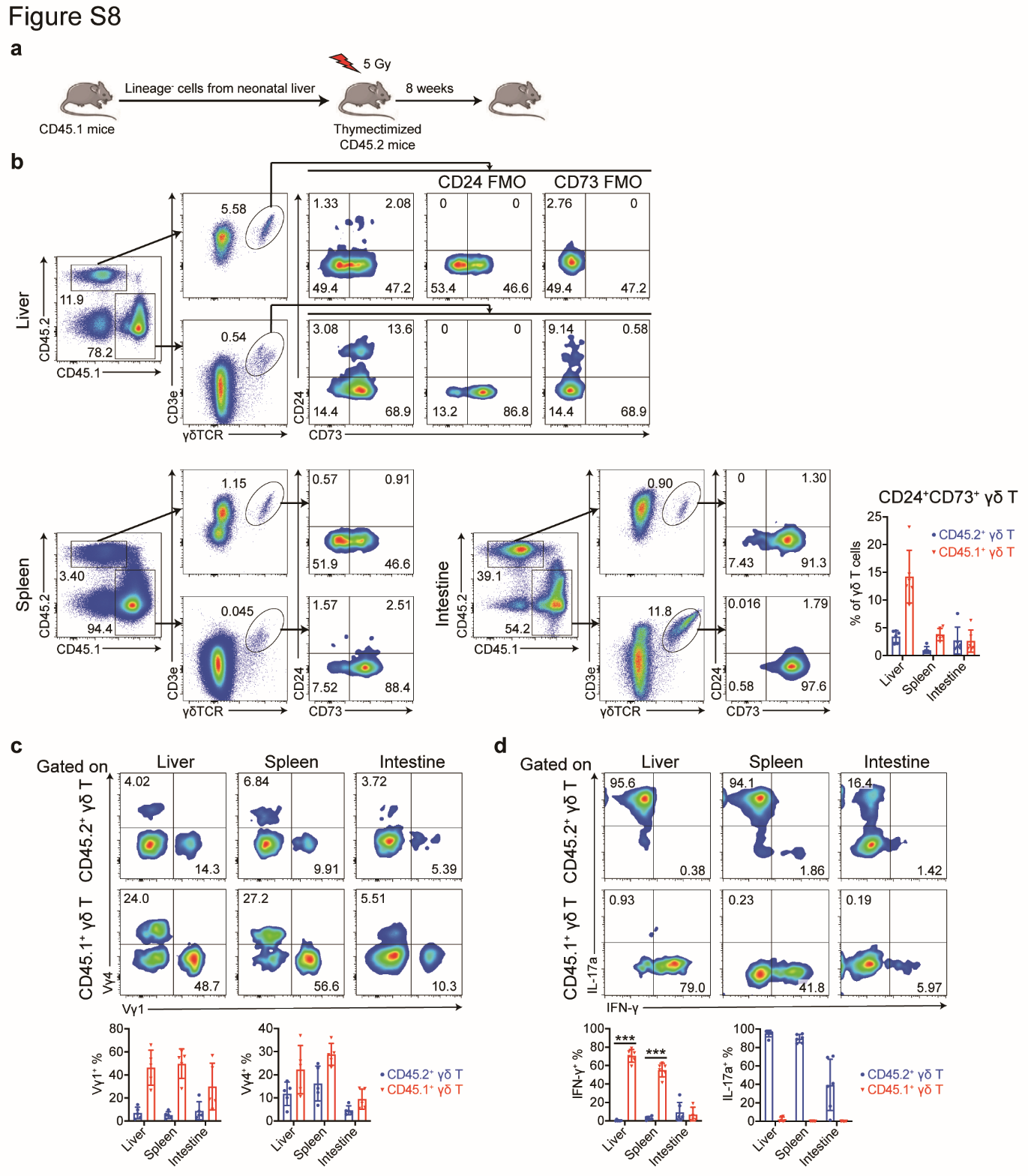
**

**Supplemental Figure S8. IFN-γ-producing γδ T cells can originate from neonatal liver Lin^−^ cells independent of the thymus. a** Schematic of the experimental design. Thymectomies were performed on 5–6-week-old *CD45.2^+^* mice. Lin^−^ cells (1 × 10^5^) sorted from neonatal livers of *CD45.1^+^* mice were intravenously transferred into 5 Gy-irradiated thymectomized *CD45.2^+^* mice. **b–d** Flow cytometry detected the expression of CD24 and CD73 to analyze the percentages of pre-γδ T cells and mature γδ T cells (**b**), the use of Vγ1 or Vγ4 chains (**c**), and the percentages of IFN-γ and IL-17a (**d**) in donor-derived or recipient γδ T cells. Statistical analysis for the percentages of IL-17a and IFN-γ in CD45.1^+^ or CD45.2^+^ γδ T cells (n = 6). All results are presented as the mean ± SEM; *, *P* < 0.05, **, *P* < 0.01, ***, *P* < 0.001 as determined by unpaired Student’s *t*-test for two-group comparisons.
